# Restoring oscillatory dynamics in Alzheimer's disease: A laminar whole-brain model of serotonergic psychedelic effects

**DOI:** 10.1162/NETN.a.540

**Published:** 2026-04-22

**Authors:** Jan C. Gendra, Edmundo Lopez-Sola, Francesca Castaldo, Èlia Lleal-Custey, Roser Sanchez-Todo, Jakub Vohryzek, Ricardo Salvador, Ralph G. Andrzejak, Giulio Ruffini, for the Alzheimer’s Disease Neuroimaging Initiative

**Affiliations:** School of Computation, Information and Technology, Technical University of Munich, Munich, Germany; Brain Modeling Department, Neuroelectrics, Barcelona, Spain; Department of Engineering, Universitat Pompeu Fabra, Barcelona, Spain; Center for Brain and Cognition, Universitat Pompeu Fabra, Barcelona, Spain

**Keywords:** Psychedelics, Alzheimer's disease, Neuropsychiatry, Serotonin, 5-HT2A, PET, Whole-brain model, Laminar neural mass model, fMRI, EEG

## Abstract

Classical serotonergic psychedelics show promise in addressing neurodegenerative disorders such as Alzheimer’s disease by modulating pathological brain dynamics. However, the precise neurobiological mechanisms underlying their effects remain elusive. This study introduces a personalized whole-brain model built upon a laminar neural mass framework to elucidate these effects. Using multimodal neuroimaging data from 30 subjects diagnosed with mild to moderate Alzheimer’s disease, we simulate the impact of serotonin 2A receptor activation, characteristic of psychedelics, on cortical dynamics. By modulating the excitability of layer 5 pyramidal neurons, our models reproduce hallmark changes in EEG power spectra observed under psychedelics, including alpha power suppression and gamma power enhancement. These spectral shifts are shown to correlate strongly with the regional distribution of serotonin 2A receptors. Furthermore, simulated EEG reveals increased complexity and entropy, suggesting restored network function. These findings underscore the potential of serotonergic psychedelics to reestablish healthy oscillatory dynamics in the prodromal and early phases of Alzheimer’s disease and offer mechanistic insights into their potential therapeutic effects in neurodegenerative disorders.

## Author Summary

Our work explores how psychedelics may help restore healthy brain function in neurodegenerative disorders such as Alzheimer’s disease. We built whole-brain models based on a laminar neural mass framework that captured alpha and gamma oscillations in local cortical dynamics. These models were personalized using multimodal MRI data from individuals with Alzheimer’s disease. The effects of psychedelics were simulated using serotonin 2A receptor density maps derived from positron emission tomography imaging. By incorporating these mechanisms, the models reproduced hallmark spectral features of psychedelics, including alpha suppression, gamma enhancement, and increased signal complexity. Our findings suggest that serotonergic psychedelics can rebalance oscillatory activity and partially restore network flexibility in the early stages of Alzheimer’s disease.

## INTRODUCTION

In the last couple of decades, classical serotonergic psychedelics (hereby, *psychedelics*) have re-emerged as potential treatments for a range of different neuropsychiatric disorders, including depression, anxiety, and posttraumatic stress disorder, yielding promising results (Cameron et al., 2023; Husain et al., 2023; Nutt, Erritzoe, & Carhart-Harris, 2020). Emerging evidence also points to the potential of psychedelics in treating neurodegenerative conditions, such as Alzheimer's disease (AD) and related dementias (Garcia-Romeu, Darcy, Jackson, White, & Rosenberg, 2022; Kozlowska, Nichols, Wiatr, & Figiel, 2022; Vann Jones & O’Kelly, 2020; Winkelman, Szabo, & Frecska, 2023). While current evidence is primarily preclinical, there is growing interest in investigating psychedelics as potential treatments for AD, with both high-dose and micro-dosing regimens being considered, and converging data suggest that psychedelics may exert beneficial effects by promoting neuroplasticity, reducing neuroinflammation, and modulating large-scale brain connectivity (Garcia-Romeu et al., 2022; Vann Jones & O’Kelly, 2020; Winkelman et al., 2023).

Serotonergic psychedelics primarily act as agonists of the serotonin 2A receptor (5-HT2AR; Cameron et al., 2023; Nutt et al., 2020), though other receptors, such as 5-HT1AR, may also contribute to these effects (Hansen et al., 2022; Warren et al., 2024). The intensity of these effects is linked to the degree of occupancy of these receptors (Madsen et al., 2019). Among the different serotonin receptor (5-HT) subtypes, 5-HT2ARs are the most abundant in the cortex (Luppi et al., 2024) and show some regional heterogeneity (Andrade, 2011; Berger, Gray, & Roth, 2009; Nutt et al., 2020). 5-HT2A receptors modulate the excitatory effects of glutamate, particularly by enhancing the excitability of deep-layer pyramidal neurons to glutamate inputs (i.e., making them more susceptible to excitatory inputs typically associated with glutamate receptors; Andrade, 2011). This enhanced excitation dysregulates the spontaneous activity in cortical populations, increasing the entropy or complexity of ongoing brain activity and producing alterations in its power spectrum (Carhart-Harris & Friston, 2019; Nutt et al., 2020). In particular, the power changes associated with 5-HT2AR activation disrupt the brain's normal alpha rhythm dynamics, resulting in a widespread decrease in the mean power spectral density (PSD) of the alpha band in the cortex (Carhart-Harris & Friston, 2019; Carhart-Harris et al., 2014; Nutt et al., 2020; Ort, Smallridge, et al., 2023; Timmermann et al., 2023) and an increase in the cortical gamma band power (Smausz, Neill, & Gigg, 2022; Timmermann et al., 2023).

These mechanisms may be particularly valuable in the context of AD, where disrupted oscillatory patterns, reduced complexity, and impaired connectivity are hallmarks of the disorder (Abásolo, Hornero, Espino, Alvarez, & Poza, 2006; Babiloni et al., 2020; Palop & Mucke, 2016). Specifically, these disruptions evolve over years across the stages of the disease, reflecting progressive impairments in neural dynamics. During the preclinical phase, alpha activity is disrupted (Gallego-Rudolf, Wiesman, Pichet Binette, Villeneuve, & Baillet, 2024; Nakamura et al., 2018), while gamma power shows variability, with reports of increases, stability, or reductions (Gaubert et al., 2019; Rochart, Liu, Fonteh, Harrington, & Arakaki, 2020). As the disease progresses into the prodromal phase, alpha power remains elevated (Nakamura et al., 2018). During this phase, gamma power is thought to decline due to the dysfunction of parvalbumin (PV) interneurons, which are essential for generating gamma oscillations and maintaining network balance (Murty et al., 2021; Palop & Mucke, 2016). In the mild to moderate stages, alpha power declines, gamma power becomes markedly reduced, and neural complexity decreases, reflecting growing impairments in synaptic function, network flexibility, and cognitive processing (Abásolo et al., 2006; Garcés et al., 2013; Sun et al., 2020). For a more detailed discussion of AD physiopathology and oscillatory deficits, see Supporting Information S1.

Psychedelics, acting through the mechanisms described above, could counteract these deficits by reducing alpha power, thereby alleviating alpha band hypersynchrony and enhancing network flexibility. Their ability to increase gamma power is particularly relevant for addressing PV interneuron dysfunction, potentially restoring gamma oscillations and rebalancing excitatory–inhibitory networks (Halberstadt, 2015; Palop & Mucke, 2016). Additionally, psychedelics enhance neural complexity, increasing entropy and promoting a more flexible and integrated network state (Carhart-Harris, 2018; Nutt et al., 2020; Ruffini et al., 2023), which may help counteract the rigidity and loss of complexity observed in AD (Abásolo et al., 2006; Simons & Abásolo, 2017; B. Wang et al., 2017). These effects suggest that psychedelics could be especially effective in the prodromal and mild stages of the disease, where compensatory mechanisms remain active, and neural circuits are less extensively damaged. A summary of how psychedelics may compensate for these deficits in AD is presented in Table 1.

**Table 1.** Compensation of AD deficits by serotonergic psychedelics, suggesting potential therapeutic value in early AD.

**Feature**	**AD deficit**	**Compensation by psychedelics**
**Alpha power**	Increased alpha power and alpha hypersynchronization (preclinical and prodromal; Gallego-Rudolf et al., 2024; López et al., 2014; Nakamura et al., 2018); eventual decrease in alpha peak frequency and alpha power in later stages (Benwell et al., 2020; Passero, Rocchi, Vatti, Burgalassi, & Battistini, 1995; R. Wang et al., 2015).	Psychedelics reduce alpha power, increasing network flexibility, and interconnectivity (Carhart-Harris & Friston, 2019; Kometer, Schmidt, Jäncke, & Vollenweider, 2013; Nutt et al., 2020). Potentially valuable in early AD.
**Gamma power**	Possible preclinical increases in gamma (Gaubert et al., 2019), decreased gamma activity in prodromal and later stages (Murty et al., 2021), impairing cognitive processes and memory (Palop & Mucke, 2016).	Psychedelics increase gamma power, promoting oscillations associated with cognitive processing (Kometer et al., 2013; Smausz et al., 2022; Timmermann et al., 2023). Potentially valuable after the preclinical phase.
**Delta/theta power**	Increased power in delta and theta bands; hypersynchrony in slow oscillations (Chetty et al., 2024; Nakamura et al., 2018; Passero et al., 1995).	Psychedelics reduce delta and theta powers, countering hypersynchrony and restoring balance across frequencies (Pallavicini et al., 2019).
**Complexity**	Decreased entropy and complexity of brain activity (Abásolo et al., 2006; Simons & Abásolo, 2017; B. Wang et al., 2017).	Psychedelics increase entropy and brain signal complexity (R. L. Carhart-Harris, 2018; R. L. Carhart-Harris & Friston, 2019; Nutt et al., 2020; Ruffini et al., 2023).
**Inhibitory-excitatory balance**	Impaired PV interneuron function disrupts gamma oscillations and leads to network instability (Palop & Mucke, 2016).	Psychedelics engage 5-HT2A receptors on pyramidal neurons, indirectly improving PV interneuron function and rebalancing excitatory–inhibitory networks (Halberstadt, 2015).
**Network features**	Decreased global connectivity and increased modularity; network decoupling (Babiloni et al., 2020).	Psychedelics increase global FC and reduce modularity, promoting integration and flexibility (Tagliazucchi et al., 2016).

A deeper understanding of the mechanisms underlying the effects of psychedelics is essential to develop such effective treatments (Cameron et al., 2023; Carhart-Harris & Friston, 2019; Nutt et al., 2020). Psychedelics affect brain dynamics across scales, from subcellular mechanisms to whole-brain networks, making computational models valuable tools for exploring their therapeutic potential. Such mechanistic models have recently proven helpful in advancing our understanding of the mechanism of action of psychedelics (Deco et al., 2018; Herzog et al., 2023; Jobst et al., 2021; Kringelbach et al., 2020; Luppi et al., 2022; Mindlin et al., 2024; Vohryzek et al., 2024; Vohryzek, Cabral, Vuust, Deco, & Kringelbach, 2022). However, as reflected in these studies, existing computational approaches to psychedelics primarily focus on macroscale brain dynamics, overlooking critical mesoscale and microscale dynamics, such as neuronal population interactions that mediate oscillatory changes. Moreover, there is a notable lack of computational studies examining the effects of psychedelics in the context of AD.

Mesoscale modeling may be well suited to reproduce the oscillatory alterations induced by psychedelics in AD, as it represents an intermediate level between the macro- and microscale and focuses on neuronal populations and local circuits within brain regions. Here, we use the term *mesoscale* to refer to local population and laminar circuit dynamics within each cortical region, in contrast to *macroscale* network interactions between regions and *microscale* single-cell mechanisms. Computational approaches such as neural mass models (NMMs) and neural field models are frequently used in mesoscale modeling to simulate the collective behavior of neuronal populations (Cook, Peterson, Woldman, & Terry, 2022; Jansen & Rit, 1995). The laminar NMM (LaNMM) framework is a natural extension of the neural mass formalism to produce coupled fast (gamma) and slow (alpha) oscillatory activity and simulate realistic electrophysiological signals (Ruffini et al., 2020; Sanchez-Todo et al., 2023). It has been recently applied in the context of AD (Sanchez-Todo, Lopez-Sola, Mercadal, & Ruffini, 2024), showing how disrupted PV-to-pyramidal connectivity can explain the PSD alterations observed in the process of neurodegeneration in AD. Additionally, we have used the LaNMM to investigate the role of cross-frequency coupling in hierarchical predictive coding, showing how error evaluation and precision mechanisms deteriorate in AD due to disruptions of inhibitory interneuron function that impair the computation and gating of prediction errors (Ruffini et al., 2025). By capturing multiband oscillatory dynamics, the LaNMM provides a unique framework for modeling neuronal processes in conditions like AD or under exposure to psychedelics, where disruptions in slow and fast oscillatory activity are prominent.

Despite increasing experimental and theoretical interest, no previous modeling work has systematically explored how serotonergic modulation may restore the oscillatory and dynamical deficits characteristic of AD. This gap motivates the present study, which aims to bridge neurobiological evidence and whole-brain computational modeling to clarify the potential mechanisms through which psychedelics could rebalance cortical dynamics in AD. Specifically, we use multimodal data from a cohort of AD subjects to develop personalized hybrid brain models based on the LaNMM. We hypothesize that these whole-brain models can reproduce the spectral changes observed in empirical studies of psychedelic administration, specifically a pronounced decrease in alpha power and an increase in gamma power. Given the known disruptions in oscillatory activity in AD, we hypothesize that psychedelics may counteract key spectral abnormalities associated with the disease—particularly the imbalance between alpha and gamma oscillations. Furthermore, we predict that these changes will correlate with the spatial distribution of 5-HT2A receptor expression, supporting the notion that psychedelics modulate cortical excitability in a regionally specific manner. Finally, we explore whether psychedelics enhance neural complexity and entropy, which have been proposed as markers of cognitive flexibility and may contribute to their therapeutic effects.

## METHODS

**Figure 1.**
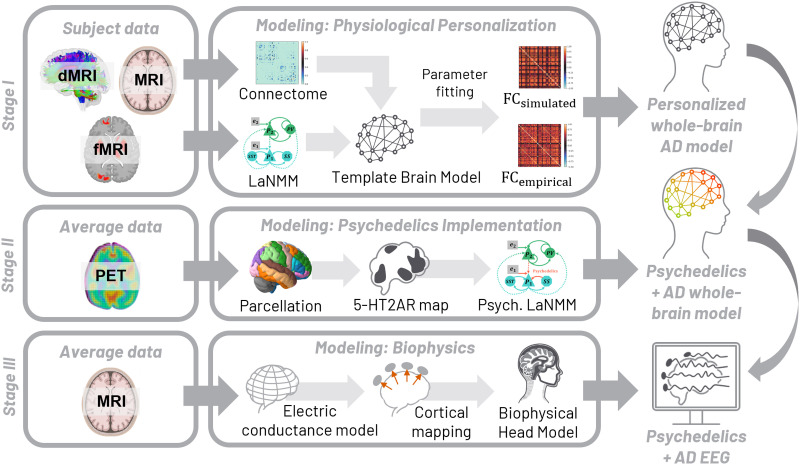
Overview of the methodology. Pipeline for personalization of hybrid brain models of AD and the effects of psychedelics using structural MRI, dMRI, fMRI, and PET data. In Stage I, we generate personalized whole-brain models based on the LaNMM using multimodal data from a cohort of AD patients. In Stage II, we simulated the effects of psychedelics in the personalized models using the distribution of 5-HT2A receptors obtained from PET, we and evaluated the oscillatory changes at the cortical level. In Stage III, we combined the whole-brain models with a biophysical head model to study the effects of psychedelics at the EEG level, including oscillatory changes and variations in complexity.

An overview of the methodology followed in this study is shown in Figure 1. We first generated subject-specific whole-brain models based on diffusion MRI (dMRI) and functional MRI (fMRI) data from AD subjects (Stage I). We then integrated the effects of psychedelics following a positron emission tomography (PET)-informed distribution of 5-HT2AR densities and studied the effects of psychedelics at the whole-brain level in the cohort of AD subjects (Stage II). We used such models to evaluate the changes in oscillatory activity at the cortical level. Finally, we combined such whole-brain models with a biophysical layer to simulate realistic electrophysiological activity (i.e., EEG) under psychedelics (Stage III) and evaluate changes in alpha and gamma power and complexity metrics.

### LaNMM Implementation

#### Model description.

The LaNMM consists of two coupled neural masses—a Jansen-Rit (JR; Jansen & Rit, 1995) and a pyramidal-interneuron-gamma (PING) model (Börgers, Epstein, & Kopell, 2008)—to simulate both slow and fast oscillations in a cortical column (Sanchez-Todo et al., 2023). The JR NMM model can generate slow oscillations in the alpha band (8–12 Hz) and is adjusted to oscillate at 10 Hz at baseline conditions. A variation of the PING model modified to oscillate at 40 Hz at baseline conditions produces fast oscillations in the gamma band (30–70 Hz).

**Figure 2.**
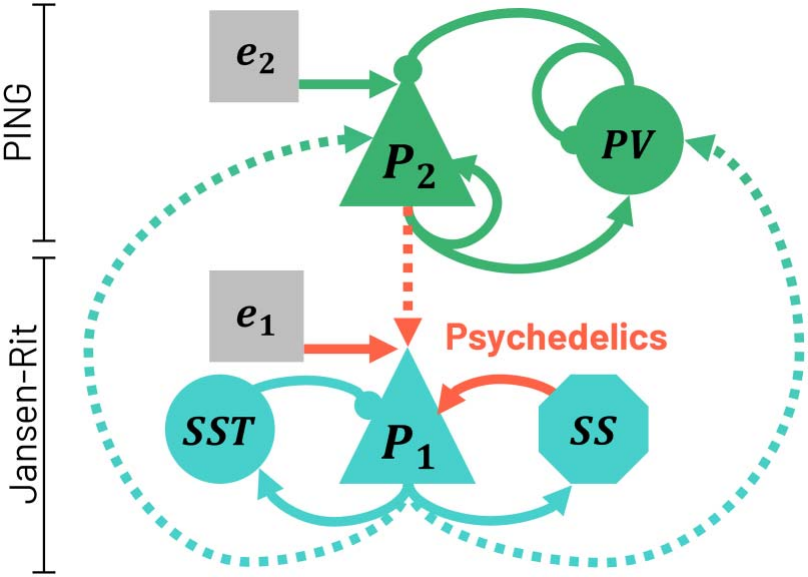
Schematic diagram of the LaNMM depicting its neuronal populations and the connections between them. Red arrows indicate the synapses affected by 5-HT2AR activation due to psychedelics in the model. Dashed lines represent connections between the PING and JR parts subcircuits.

The JR subcircuit (Figure 2) consists of a population of pyramidal neurons (representing layer 5 pyramidal cells; *P*_1_) coupled via excitatory connections to a population of excitatory neurons (representing mostly spiny stellate neurons: *SS*) and to a population of slow inhibitory interneurons (representing somatostatin-expressing cells: *SST*). Both *SS* and *SST* populations are reciprocally coupled to *P*_1_ through excitatory and inhibitory synapses, respectively. On the other hand, the PING subcircuit consists of a population of pyramidal cells (representing layer 2/3 pyramidal cells: *P*_2_) and a population of fast inhibitory interneurons (representing PV-positive cells).

External perturbations *e*_1_ and *e*_2_ are applied to *P*_1_ and *P*_2_, respectively, to represent the influence of other brain areas. The PING and JR subcircuits are reciprocally coupled through excitatory connections between the pyramidal populations *P*_1_ and *P*_2_ and through the PING's inhibitory population, thus constituting a single NMM. A schematic diagram can be found in Figure 2.

#### LaNMM equations.

The LaNMM employs a second-order differential equation to model the average membrane potential perturbation in a neuronal population *m* caused by inputs from another population *n*. This equation captures the synaptic dynamics within the average neuron of a given population, translating the presynaptic mean firing rate *φ_n_* into a perturbation of the mean membrane potential *u*_*m*←*n*_ in the postsynaptic population *m*. This relationship is expressed through the following equation:

$${\hat{L}}_{m\leftarrow n}\left[u_{m\leftarrow n}\left(t\right)\right]=C_{m\leftarrow n}\varphi n\left(t\right)$$ (1)

where *C*_*m*←*n*_ is the connectivity constant, indicating the average number of synaptic contacts between population types, and ${\hat{L}}_{m\leftarrow n}$ is a differential operator defined as follows:

$${\hat{L}}_{s}\left[u_{s}\left(t\right)\right]=\frac{1}{A_{s}}\left(\frac{1}{a_{s}}\frac{d^{2}}{{dt}^{2}}+2\frac{d}{dt}+a_{s}\right)u_{s}\left(t\right)$$ (2)

where *A*_*s*_ is the average excitatory/inhibitory synaptic gain and *a*_*s*_ is the rate constant of the connection (*a*_*s*_ = 1/*τ_s_, τ_s_* denoting the synaptic time constant). Note that, for simplicity, the single index *s* is used to represent each average synapse (i.e., connection) from one neuronal population to another, so that *s* ≡ {*m* ← *n*:*C*_*m*←*n*_ ≠ 0}. In the LaNMM, *s* ∈ [1, 13] and the neural populations are denoted by (*m*, *n*) ∈ [*P*_1_, *SS*, *SST*, *P*_2_, *PV*, *e*1, *e*2]. The indices assigned to each connection in the LaNMM are depicted in Supporting Information Figure S1a. Finally, the average output firing rate *φ_m_* of population *m* is defined by the following sigmoid function:

$$\varphi_{m}\left(t\right)=\sigma_{m}\left(\nu_{m}\left(t\right)\right)=\frac{2\varphi_{0}}{1+e^{r\left(\nu_{0}-\nu_{m}\left(t\right)\right)}}$$ (3)

where *φ*_0_ is half of the maximum firing rate of the population, *ν*_0_ is the value of the potential when the firing rate is *φ*_0_, and *r* determines the slope of the sigmoid at the central symmetry point (*ν*_0_, *φ*_0_). Here, *ν_m_* denotes the average membrane potential of the postsynaptic population *m*:

$$\nu_{m}\left(t\right)=\sum_{s}u_{s}\left(t\right)$$ (4)

where *u*_*s*_ is the membrane perturbation for each of its incoming connections *s*. This modeling approach focuses on the dynamics of the connections between neural populations instead of the populations' dynamics, as opposed to the traditional approach followed in neural mass modeling. This is referred to by Sanchez-Todo et al. (2023) as the *synapse-driven* formulation of an NMM, which simplifies the definition of the neural dynamics generalizing the model equations. The model parameters used are shown in Supporting Information Table S1.

#### Implementation of psychedelics effects.

In line with experimental evidence, we modeled the effect of psychedelics as acting selectively on layer 5 pyramidal neurons (*P*_1_), which are the primary cortical targets of the 5-HT2A receptor modulation (Nichols, 2016). The increased excitability to glutamate inputs observed under psychedelics can be implemented in the model as an increase of the average synaptic gain *A*_*L*5*P*_ parameter of excitatory connections to L5 pyramidal populations in the LaNMM. This parameter determines the extent to which the average firing rate of a population of neurons increases or decreases in response to an input membrane potential perturbation. Three glutamatergic connections to L5 pyramidal neurons are present in the LaNMM: connections from the *SS* and *P*_2_ populations to *P*_1_, and external noise perturbation *e*_1_ to *P*_1_. Hence, we model the 5-HT2AR activation by transforming receptor stimulation into changes of the excitatory synaptic gain *A*_*L*5*P*_ in those connections.

We studied the dynamics of a single uncoupled LaNMM for different *A*_*L*5*P*_ values in the three targeted connections. For simplicity, we modified this parameter equally for all these synapses, assuming that the activation of 5-HT2ARs affects similarly all L5 pyramidal synapses in a particular cortical column. We have used the average membrane potential activity of the pyramidal populations to study the dynamics of the uncoupled LaNMM, given that pyramidal neurons are thought to be the main contributors to the potential recorded in electrophysiological signals such as EEG (Buzsáki, Anastassiou, & Koch, 2012; Nunez & Srinivasan, 2006).

The model was simulated for each gain value *A*_*L*5*P*_ ∈ [0, 10] mV in steps of 0.05 mV. For each simulation, *A*_*L*5*P*_ was simultaneously and equally increased in all three target connections, and the LaNMM was run for 40 s with a sampling frequency *f*_*s*_ = 1,000 Hz. We chose to simulate the model for 40 s to allow a conservative 10-s transient period (well above the slowest neural time constants) and to provide a 30-s steady-state record long enough for low-variance spectral estimation. The PSD of the membrane potential of each pyramidal population *P*_1_ and *P*_2_ was computed over the last 30 s of simulation using Welch's method (P. Welch, 1967) with a segment length of 1,000 samples.

### AD Whole-Brain Models Personalization

We created personalized whole-brain models of a cohort of 30 AD subjects using the LaNMM (Stage I in Figure 1).

#### Patient data.

We used data from 30 AD subjects from the Alzheimer's Disease Neuroimaging Initiative 3 (ADNI3) database (https://adni.loni.usc.edu/). The ADNI was a multicenter study initiated by Michael W. Weiner in 2004, which focused on the early diagnosis and follow-up of AD. We selected subjects diagnosed with AD for whom the multimodal data required was available and with sufficient variability in age, gender, and cognitive function. The cohort selected consisted of 13 women and 17 men, with a mean age of 73.6 years (*SD* = 7.32). The average Mini-Mental State Examination score, a standard measure of cognitive function, ranged from 17 to 30 (mean = 22.5, *SD* = 2.7), reflecting variability in the extent of cognitive decline among the subjects selected. The data acquisition parameters for each modality used in this study and the patient cohort selected are provided in Supporting Information S3.

The sample size of 30 subjects (28 after exclusion, see in the Results section the Whole-Brain Model Personalization section) is consistent with previous modeling studies of psychedelics, which analyzed between 9 and 20 subjects (Deco et al., 2018; Luppi et al., 2022; Mindlin et al., 2024; Ruffini et al., 2023; Vohryzek et al., 2024). Experimental studies investigating PSD and neural complexity and entropy alterations under serotonergic psychedelics report robust neural effects with up to 15 participants for Lysergic Acid Diethylamide (LSD), 22 for psilocybin, and 20 for N,N-dimethyltryptamine (DMT; Carhart-Harris et al., 2014; Muthukumaraswamy et al., 2013; Ort et al., 2023; Singleton et al., 2022; Timmermann et al., 2023).

#### Data processing.

Each modality underwent a dedicated processing pipeline to generate subject-specific inputs for the whole-brain models. T1-weighted MRI data were used to create a subject-specific Desikan–Killiany (DK-68) cortical atlas (Desikan et al., 2006). The MRI processing pipeline, based on FreeSurfer (Fischl et al., 2004), began with cortical reconstruction using the *recon-all* function to extract the pial surface. The DK-68 parcellation, provided by FreeSurfer, was mapped to the subject-specific brain structure. This volumetric image served as the basis for processing dMRI and resting-state fMRI (rs-fMRI) data.

dMRI data were processed to calculate the subject's structural connectome (SC). Preprocessing steps included denoising, artifact correction (e.g., eddy currents), and estimating the fiber orientation density using constrained spherical deconvolution. Probabilistic tractography was then performed with MRtrix3 (Tournier et al., 2019), generating 20 million streamlines constrained to start and terminate in gray matter parcels defined by the MRI parcellation. The resulting structural connectome matrix quantified the number of streamlines connecting each pair of parcels and was normalized by the total number of fibers and the number of parcels to generate a connectivity matrix for the subject.

BOLD rs-fMRI data were preprocessed using fMRIPrep 22.0.0 (Esteban et al., 2019), which utilizes Nipype 1.8.3 (Gorgolewski et al., 2011). For anatomical data, T1-weighted images were corrected for intensity nonuniformity (N4BiasFieldCorrection) and skull-stripped using Advanced Normalization Tools (ANTs; Avants, Tustison, & Song, 2009). Tissue segmentation was performed with FSL FMRIB’s Automated Segmentation Tool (FAST), and brain surfaces were reconstructed with FreeSurfer. Functional data preprocessing included motion correction (FSL mcflirt), slice-timing correction (Analysis of Functional NeuroImages (AFNI) 3dTshift), coregistration to T1-weighted images (FreeSurfer bbregister), and normalization to MNI templates using ANTs. Confound regressors, including motion parameters, CompCor components, and framewise displacement, were calculated to facilitate nuisance regression. Detailed preprocessing workflows and parameters are described in Supporting Information and in the fMRIPrep documentation (https://fmriprep.org).

#### Model fitting.

In our framework, whole-brain models represent the human cortex as a network of coupled NMMs, with each node representing the average activity of a cortical parcel through a LaNMM and each link between them modeling the white matter fibers connecting these parcels. The connectome (obtained from dMRI data as described above) was used to provide the connectivity between nodes. *P*_1_ and *P*_2_ populations of different parcels *i, j* were connected as follows: $P_{1}^{i}\to P_{1}^{j}$ (lateral connection), $P_{1}^{i}\to P_{2}^{j}$ (feedback or descending connection), and $P_{2}^{i}\to P_{2}^{j}$ (feedforward or ascending connection) with relative weights of 0.5, 0.5, and 1 respectively. Further details about the long-range connectivity are given in Supporting Information S2.2.

Whole-brain models were personalized to match each subject's empirical functional connectivity (FC) obtained from their BOLD rs-fMRI, ultimately tailoring the models to reflect individual neural dynamics. Synthetic BOLD data from the whole-brain models were generated using the Balloon–Windkessel model (Friston, Mechelli, Turner, & Price, 2000), and FC matrices were computed using Pearson's correlation coefficient (PCC) between the BOLD signals. Each whole-brain model was simulated for five different noise realizations for a duration of 300 s with a sampling frequency of *f*_*s*_ = 1,000 Hz. The first 5 s of the simulation were discarded to account for transient dynamics and ensure the models reached a steady state. The fourth-order Runge–Kutta method (Press & Teukolsky, 1992; RK4) was used to solve the model's differential equations.

Model optimization involved tuning the global coupling gain *G* (which scales the connectome) and the noise received by the pyramidal populations of each region (in particular, the ratio between common and homotopic noise standard deviations [*SD*s] *σ_c_*/*σ_i_*; see Supporting Information S2.3). A grid search was performed to select the optimal values of *G* and noise that minimized the root mean squared error (RMSE) between simulated and empirical FC matrices for each subject. The best value across noise realization was selected. An additional constraint was applied, requiring the PCC between simulated and empirical FC matrices to be higher than the PCC between the empirical FC and the structural connectivity matrix. This ensured that the models captured FC patterns beyond those explained by anatomical connectivity.

### Whole-Brain Models Under Psychedelics

The 30 subject-specific whole-brain models described in the Methods section were adapted to reproduce the activation of 5-HT2A receptors (Stage II in Figure 1). Following the approach described in the previous sections, we implemented the activation of 5-HT2A receptors as changes in the synaptic gain of all excitatory inputs to *P*_1_ pyramidal population (*A*_*L*5*P*_) in every LaNMM of the brain network. This includes connections from the *SS* and *P*_2_ populations to *P*_1_, the common and homotopic external inputs (see Supporting Information S2.3), and all *P*_1_ → *P*_1_ long-range corticocortical inputs.

To modulate the average synaptic gain of the connections involved in the psychedelic effect, we used 5-HT2A receptor density data informed by the high-resolution *in vivo* atlas of the human brain serotonin system measured with PET in Beliveau et al. (2017). We refer the reader to that paper for further details on the PET and MRI acquisition parameters and the participant's demographics. We parcellated the 5-HT2AR average density map into the DK-68 cortical atlas as detailed in Supporting Information S4. We then normalized the density values *B*_*max*_ to fall between 0 and 1. This provides us with the relative spatial distribution of 5-HT2A receptors between brain areas, defining the amount of serotonergic stimulation each brain area experiences under psychedelic drugs.

To translate the receptor density map into average synaptic amplitude gain values of glutamatergic connections to *P*_1_ (*A*_*L*5*P*_), we define the neuromodulation relation

$$A_{L5P}^{i}=A_{0}+\psi \cdot R_{i}\cdot \left(A_{\max}-A_{0}\right)$$ (5)

where ψ is the psychedelic dose (0 at baseline, 1 under psychedelics) and *R*_*i*_ ∈ [0, 1] is the normalized parcel's receptor density. *A*_0_ = 3.25 mV is the nominal gain, that is, the average synaptic amplitude gain of pyramidal neurons at baseline conditions (Jansen & Rit, 1995). *A*_*max*_ = 3.75 mV is the maximum gain, that is, the gain at which the PSD decay of the alpha band in the *P*_1_ population ceases to be linear when increasing *A*_*L*5*P*_ in an uncoupled LaNMM. Hence, this maps the receptor densities between *A*_0_ and *A*_*max*_ and maintains a linear relationship between *R* and *A*_*L*5*P*_. The normalization applied to *B*_*max*_ assumes that the minimum density of receptors corresponds to the nominal gain *A*_0_. Of note, we use the same *A*_*max*_ value in all parcels and apply the same spatial distribution of 5-HT2AR densities to each subject.

Each subject's whole-brain model of psychedelic effects was simulated for a duration of 40 s with a sampling frequency *f*_*s*_ = 1,000 Hz. The RK4 method was used to solve the model's differential equations. The first 10 s of the simulation were excluded in all subsequent analyses to account for the transient time of the models and their convergence to the steady state. We computed the parcel-averaged power spectrum of each subject's membrane potential and conducted Wilcoxon signed-ranks tests in each brain band (delta: 0.5–4 Hz, theta: 4–8 Hz, alpha: 8–12 Hz, beta: 12–30 Hz, and gamma: 30–70 Hz), comparing the mean PSD in each frequency band across subjects between the baseline and psychedelic conditions. We used the Wilcoxon signed-ranks test as a nonparametric alternative to the paired *t* test, given that the data did not conform to a normal distribution according to the Kolmogorov–Smirnov test (Bonferroni-corrected *p* < 0.05 in all frequency bands and conditions).

To assess whether the oscillatory changes correlated with the distribution of 5-HT2A receptors obtained from PET, we measured the PCC between the density of 5-HT2A receptors in each parcel and the absolute PSD difference (*PSD*_Post_ − *PSD*_Pre_) in both the alpha and gamma bands in that parcel. Parcels with ${PSD}_{P_{1}}^{\alpha }$ < ${PSD}_{\min}^{\alpha }$ at baseline were excluded from the analysis since their alpha power was not expected to show relevant variations due to changes in receptor density. Parcels with such low alpha power are spatially scattered across the cortex, and their exclusion did not introduce any spatial sampling bias.

### Simulation of EEG Recordings

We generated synthetic EEG data for each subject using *hybrid brain models*, which combine the physiological whole-brain models developed in the previous section with a biophysical head model template (Miranda, Mekonnen, Salvador, & Ruffini, 2013; Ruffini, Fox, Ripolles, Miranda, & Pascual-Leone, 2014; Ruffini et al., 2013; Stage III in Figure 1). The process of EEG generation from the simulated activity in the whole-brain model NMMs can be divided into two steps: (a) generation of the equivalent dipole strength over time from NMM activity, (b) generation of EEG signals from the dipole strength.

To translate NMM activity into the equivalent dipole strength, we used the mesoscale physical model of volume conduction and the neuron compartment models of current generation from Mercadal et al. (2023), which were developed based on the laminar framework developed in Sanchez-Todo et al. (2023). In brief, a multicompartmental modeling formalism and detailed geometrical models of pyramidal cells are used to simulate the transmembrane currents due to synaptic inputs into different cortical layers. These results are linearly combined to estimate the total current source densities (CSDs) generated due to the neural activity in pyramidal populations, which are considered the main current generators (Buzsáki et al., 2012; Nunez & Srinivasan, 2006). The CSDs are then used to calculate the equivalent dipole strength over time associated with the neural activity in each parcel of the subject-specific whole-brain model.

To generate scalp EEG signals from the dipole activity, we combined the personalized whole-brain model with a template biophysical head model, creating a hybrid brain model (Sanchez-Todo et al., 2018). Supporting Information S5 details the process of template head model creation. We used such a head model of electrical conduction to compute cortical mappers for EEG and generate the EEG signals in each electrode from the equivalent dipole strength computed in the personalized whole-brain models.

We computed the power spectrum of each EEG channel averaged across subjects. We then conducted Wilcoxon signed-ranks tests in the alpha and gamma bands, comparing the mean band PSD of both conditions. The use of the Wilcoxon signed-ranks test was again justified by the nonnormal distribution of the data, confirmed by the Kolmogorov–Smirnov test (Bonferroni-corrected *p* < 0.05 in all EEG channels and conditions).

#### Complexity and entropy of simulated EEG.

To capture the complexity of the simulated EEG signals obtained from the personalized whole-brain models under psychedelics, we used practical approximations of algorithmic complexity based on Lempel–Ziv compression and entropy rate, as detailed in Ruffini (2017) and Ruffini et al. (2019).

We estimated the *Lempel-Ziv complexity* of the EEG data signals using the Lempel–Ziv–Welch (LZW) method (Cover, 1999; Ruffini, 2017; Ruffini et al., 2019; T. A. Welch, 1984). We used a single 5-s epoch of simulated broadband EEG data (0.5–100 Hz) generated from each subject's personalized computational model for this calculation and the subsequent entropy rate analysis, as the intrasubject variability of the synthetic data was negligible. The 5-s epoch of each channel was binarized based on the median value of the epoch: Each value was converted into a “0” if it was below the median and into a “1” otherwise (Ruffini, 2017). The binarized data of all channels was concatenated, and the LZW method was applied to the resulting string. A natural way to normalize the description length resulting from the LZW method, *l*_*LZW*_, is to divide it by the original string length *n, ρ*_0_ = *l*_*LZW*_/*n*, with units of bits per character. We computed *ρ*_0_ following this method for each subject.

To complement this analysis, we computed the related *entropy rate* of the binarized EEG signals for each subject. For a stationary stochastic process {*X*_*i*_}, the entropy rate H(*X*) is defined as follows:

$$H\left(X\right)=\lim_{n\to \infty }\frac{H\left(X_{1},\ldots ,X_{n}\right)}{n}\;=\;\lim_{n\to \infty }H\left(X_{n}|X_{n-1},\ldots ,X_{1}\right),$$

where *H*_*n*_(*X*) = *H*(*X*_1_, …, *X*_*n*_) is the joint entropy and $H\left(X\right)=-E_{X}\left[\log P\left(X\right)\right]$. For a Markov process of order *n*, the entropy rate simplifies to the conditional entropy, given the previous *n* states. In the special case of *n* = 0 (an independent and identically distributed process), the entropy rate reduces to the entropy of a single symbol, *H*(*X*_*i*_). Following these definitions, we computed the entropy rate for Markov orders *n* = 0, …, 5. (Cover, 1999; Ruffini et al., 2019).

Finally, we calculated the *spectral entropy* of the 5-s epoch of simulated EEG as an alternative measure of neural entropy under baseline and psychedelic conditions. To achieve this, for each subject, the PSD was averaged across channels and normalized to sum to 1, thereby transforming it into a probability distribution function. The spectral entropy was then computed for each channel using Shannon's entropy, *H*(*p*) = − ∑*p*_*i*_ ⋅ log*p*_*i*_, where *p*_*i*_ is the PSD normalized to unit sum.

## RESULTS

### Uncoupled LaNMM Spectral Profile

We first studied the power spectrum of pyramidal populations *P*_1_ (generator of alpha oscillations) and *P*_2_ (generator of gamma oscillations) in an uncoupled LaNMM, and we assessed the LaNMM's ability to simulate the cortical spectral alterations resulting from 5-HT2AR psychedelics activation in this range.

**Figure 3.**
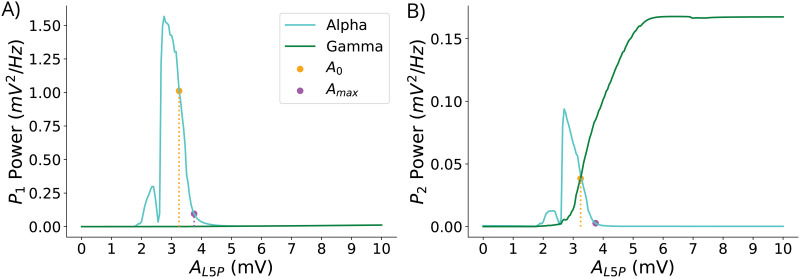
Uncoupled LaNMM spectral profile. Mean PSD of the membrane potential of *P*_1_ (A) and *P*_2_ (B) in the alpha band (blue) and gamma band (green) for different values of synaptic gain *A*_*L*5*P*_. The vertical dashed lines indicate the nominal gain *A*_0_ (orange) and the maximum gain *A*_*max*_ (purple), respectively.

Figure 3 shows the mean PSD in the alpha and gamma bands of populations *P*_1_ and *P*_2_ for different synaptic gains *A*_*L*5*P*_ ∈ [0, 10] mV. We can observe a window of synaptic gain values *A*_*L*5*P*_ ∈ [2.75, 4.15] mV for which both alpha and gamma oscillations are present in the LaNMM (as further detailed in Supporting Information S6).

The results show a main peak in the mean PSD of *P*_1_'s alpha band coinciding with the start of the gamma oscillations in *P*_2_ at *A*_*L*5*P*_ = 2.75 mV. The decay of alpha activity in *P*_1_ starts concurrently with the growth of gamma activity in *P*_2_, indicating a reciprocal and inverse relationship to synaptic gain changes between the two pyramidal populations for *A*_*L*5*P*_ ∈ [2.75, 4.15] mV. The nominal synaptic gain *A*_0_ = 3.25 mV used in Sanchez-Todo et al. (2023) and Jansen and Rit (1995) falls within this interval. This indicates that at baseline conditions (no psychedelics applied), *P*_1_ oscillates in the alpha band; and *P*_2_, in the gamma band. The decay of alpha power in *P*_1_ becomes nonlinear at around 3.75 mV, with a PSD in alpha of about 0.1 mV^2^/Hz. We will use this value as the maximum gain of the pyramidal populations in our model, *A*_*max*_ = 3.75 mV (corresponding to a minimum PSD in the alpha band of ${PSD}_{\min}^{\alpha }=0.1{mV}^{2}/Hz$).

Therefore, the decrease in alpha power in *P*_1_ and the increase in gamma power in *P*_2_ can be reproduced in the model through the selective increase of glutamatergic gain of synapses targeting L5 pyramidal neurons, associated with higher 5-HT2AR densities. The inverse relationship between alpha and gamma power is sustained within our regime of interest *A*_*L*5*P*_ ∈ [*A*_0_, *A*_*max*_]. It is worth mentioning that the most significant changes induced by psychedelics in the PSD magnitude in our LaNMM are specific to the alpha and gamma bands within the gain range under study in both the *P*_1_ and *P*_2_ populations (Supporting Information Figure S5).

### Whole-Brain Model Personalization

The personalization of the whole-brain models for the 30 AD subjects resulted in high similarity between the subjects' rs-fMRI BOLD FC and the synthetic FC obtained with the fitted model, with an average PCC across subjects of 0.45 and an average RMSE of 0.18 after model personalization.

**Figure 4.**
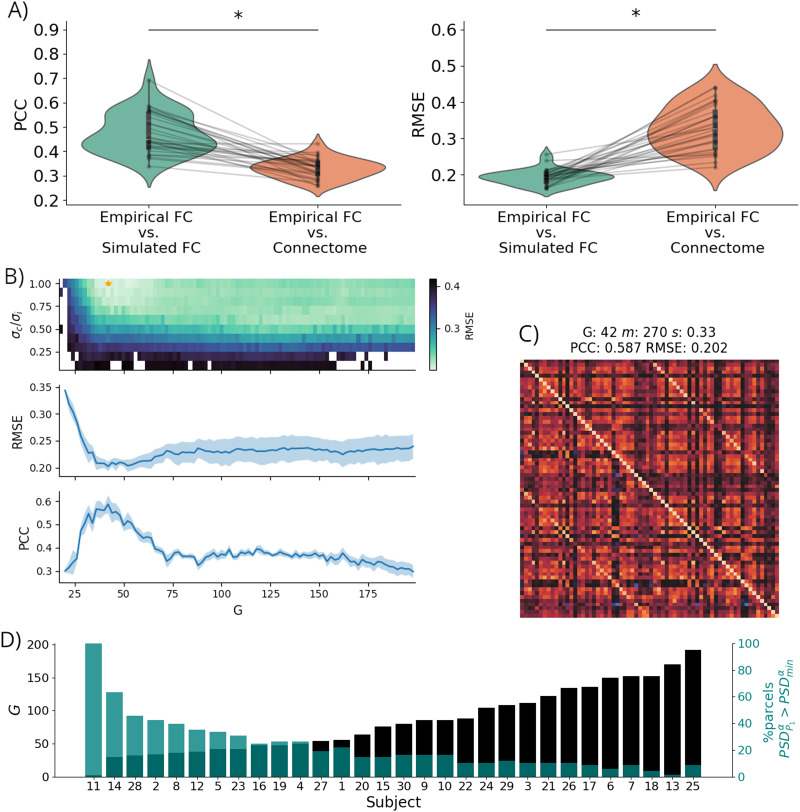
Whole-brain model fitting results. (A) PCC and RMSE between the empirical FC and simulated FC compared with the PCC and RMSE of the empirical FC and the structural connectome. Asterisks denote statistical significance in paired *t* tests. (B) Example of model personalization (subject 5): RMSE between empirical and synthetic FC for different values of the parameters *G* and *σ_c_*/*σ_i_* (top), RMSE, and PCC as a function of the G value (center and bottom). (C) For the same subject, comparison between the empirical rs-fMRI BOLD FC (lower triangular matrix) and the synthetic FC obtained with the fitted model (upper triangular matrix). (D) Optimal *G* parameter found for each subject-specific model (from lowest to highest) and number of DK-68 parcels of each model with meaningful alpha activity in *P*_1_ (${PSD}_{P_{1}}^{\alpha }>{PSD}_{\min}^{\alpha }$).

For all subjects, we found higher similarity (captured with RMSE and PCC) of the empirical FC with the model-generated FC than with the structural connectivity, confirming that the models were able to explain the functional data beyond the information provided by the subject-specific connectome alone (Figure 4A). The values of PCC, RMSE, and fitted parameters for each subject can be found in Supporting Information S7, as well as the empirical and synthetic FC matrices for each subject. We also computed the dynamic FC (dFC) of the simulated data for each subject and confirmed that the variance of dFC values across time was higher than that of randomized surrogate data, indicating the presence of nontrivial temporal fluctuations consistent with empirical fMRI dynamics (see Supporting Information S8).

As an example of model parameter optimization, Figure 4B shows the grid search performed to find the minimum value of RMSE for different combinations of *G* and *σ_c_*/*σ_i_* for one subject model personalization. The FC matrices obtained from the synthetic BOLD signal for the optimal parameter selection for this subject are shown in Figure 4C and compared with the subject's BOLD FC, showing the similarity between both matrices.

We then analyzed the optimal *G* parameter values found for each subject and their relationship to the alpha activity at baseline, which is crucial for the simulation of the effects of psychedelics in the model. Figure 4D shows the *G* parameter across subject models and the percentage of parcels in each model that are above the threshold of mean PSD for *P*_1_'s alpha band (i.e., ${PSD}_{P_{1}}^{\alpha }>{PSD}_{\min}^{\alpha }$). We ordered the values according to the magnitude of the personalized *G* parameter to facilitate the visualization. The high negative correlation observed between alpha activity and *G* (*r*_*PCC*_ = −0.80, *p* < 0.0001) evidences that personalized models with higher *G* values have less overall alpha activity in the brain. Since *G* controls the global excitation of the brain model, the higher *G* is, the more the *P*_1_ population activity approaches the region where alpha activity is lost (see Supporting Information Figure S4).

Of note, subject 11 showed an abnormally low *G*, which resulted in alpha oscillations in all cortical areas due to a globally diminished excitability. Upon deeper inspection, we noted that for this subject, the grid-search selected parameter values resulted in total disconnection between nodes, leading to nonrealistic dynamics. On the other hand, subject 13 showed an abnormally low alpha activity at baseline with only one alpha-active parcel, which is strongly related to its high *G* value. Further analysis revealed that all subjects except for subject 13 were responsive to 5-HT2AR stimulation. For these reasons, we excluded both subjects 11 and 13 from the calculations and analysis in the next sections.

It is worth mentioning that, on average, only 21% of the parcels have meaningful alpha activity at baseline (i.e., above ${PSD}_{\min}^{\alpha }$) in the personalized models. Nevertheless, the models are able to reproduce significant changes in the mean alpha band activity following the simulation of 5-HT2AR activation, as shown in the next section.

### Whole-Brain Spectral Profile Under Psychedelics

**Figure 5.**
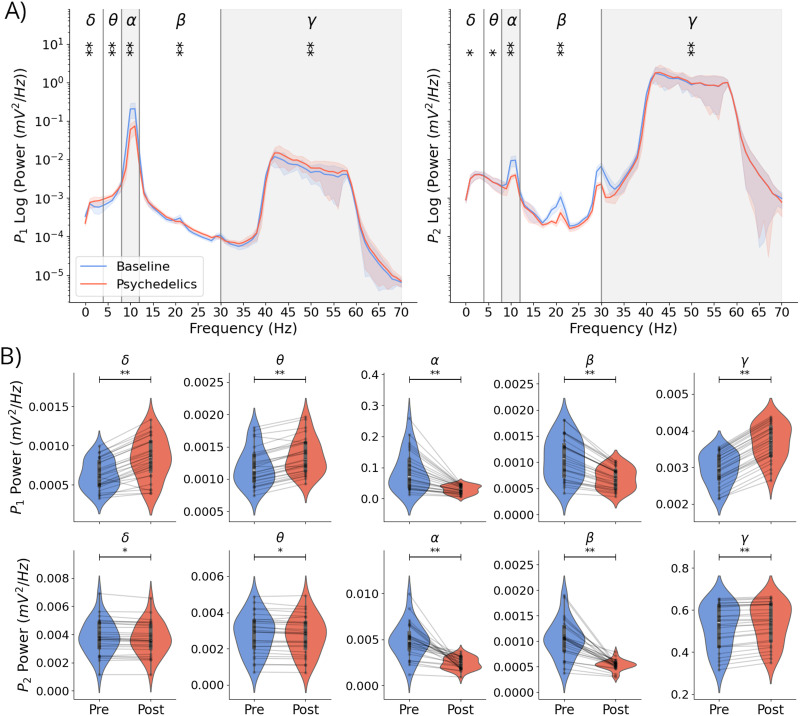
Whole-brain models reproduce the power spectrum alterations associated with psychedelics at the cortical level. (A) Power spectrum of *P*_1_ (left) and *P*_2_ (right) membrane potential averaged across brain parcels and subjects. Shaded areas represent the median absolute deviation across subjects, and solid line curves represent the average across subjects. The significance level (Bonferroni-corrected) of the average PSD difference between conditions in each band is denoted by the number of asterisks (*p < 0.05, **p < 0.0001). (B) Parcel-averaged membrane potential PSD of *P*_1_ (top) and *P*_2_ (bottom) in different bands across subjects. Black bars represent the interquartile range. Individual dots connected by gray lines represent the mean power for each subject. Asterisks denote statistical significance as described above.

Next, we studied the power spectrum of the AD whole-brain models and the spectral changes caused by psychedelics at the brain network level. Figure 5A displays the power spectrum in *P*_1_ and *P*_2_ averaged across parcels and subjects and the results of the Wilcoxon signed-ranks test. Consistent with our findings for the uncoupled LaNMM, clear peaks are observed in the alpha and gamma bands in the baseline condition. Moreover, the statistical analyses revealed a significant decrease in mean alpha power in both *P*_1_ (−61%) and *P*_2_ (−44%) and a significant increase in mean gamma power in both *P*_1_ (−25%) and *P*_2_ (−3.5%). The level of significance across tests was Bonferroni-corrected. The tendency toward a decrease of alpha power and an increase of gamma power in both pyramidal populations was consistently observed in all subjects, as shown in Figure 5B.

Although our model was specifically designed to produce alpha and gamma oscillations, we observed significant alterations across all bands and pyramidal populations under psychedelic conditions. Alpha and beta oscillations decreased on average with the 5-HT2AR stimulation, while gamma oscillations increased. Nevertheless, the delta and theta bands showed discrepancies between the two pyramidal populations since their mean power consistently increased in *P*_1_ but decreased in *P*_2_ in every subject. The large standard deviation seen for the alpha band is consistent with the high variability seen in alpha power and *G* values between different subjects in Figure 4D.

Alterations in the beta band result from the harmonics of alpha oscillations (see Supporting Information Figure S5), while changes in the theta and delta frequency bands mainly result from the external noisy input introduced to population *P*_1_, a connection also susceptible to 5-HT2AR stimulation. The frequency power analysis also highlights that the greatest magnitude change in PSD is specific to the alpha and gamma frequency bands, which are the main focus of our study.

### Influence of 5-HT2AR Distribution on Oscillatory Activity

**Figure 6.**
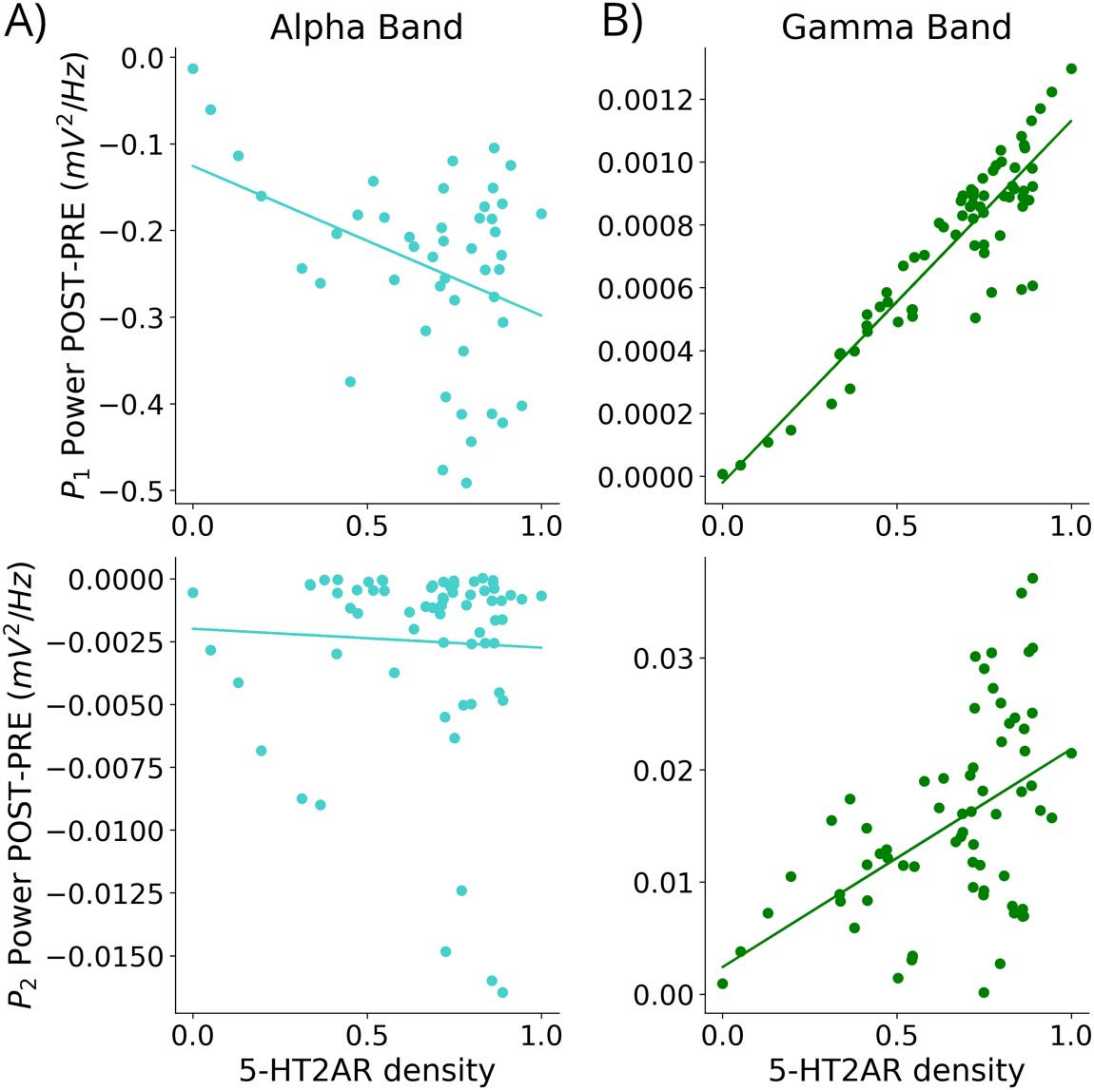
Spectral alterations under psychedelics reflect the spatial distribution and density of 5-HT2A receptors. Subject averaged parcel alpha band (A) and gamma band (B) membrane potential PSD difference (*PSD*_Post_ − *PSD*_Pre_) against parcel 5-HT2AR density in *P*_1_ (top) and *P*_2_ (bottom). Each dot represents a parcel, and solid lines are showing linear regression fits.

The changes in the spatial distribution of alpha and gamma activity across the brain are expected to relate to the applied PET-informed distribution of neurotransmitter densities. To evaluate this, we measured the PCC between the parcels' density of 5-HT2A receptors and their absolute PSD difference (*PSD*_Post_ − *PSD*_Pre_) in both the alpha and gamma bands. Figure 6 shows the results of the analysis averaged across subjects (i.e., PSD difference of the parcel averaged across subjects). We found strong correlations for both the alpha band (*P*_1_ *r*_*PCC*_ = −0.38, *p* < 0.009) and the gamma band (*P*_1_ *r*_*PCC*_ = 0.91, *p* < 0.0001; *P*_2_ *r*_*PCC*_ = 0.49, *p* < 0.0001), except for the alpha power in *P*_2_ (*r*_*PCC*_ = −0.04, *p* = 0.7). This suggests that regions with the highest expression of 5-HT2A receptors were most affected by psychedelics in the model.

### Model-Generated EEG Alpha and Gamma Power

**Figure 7.**
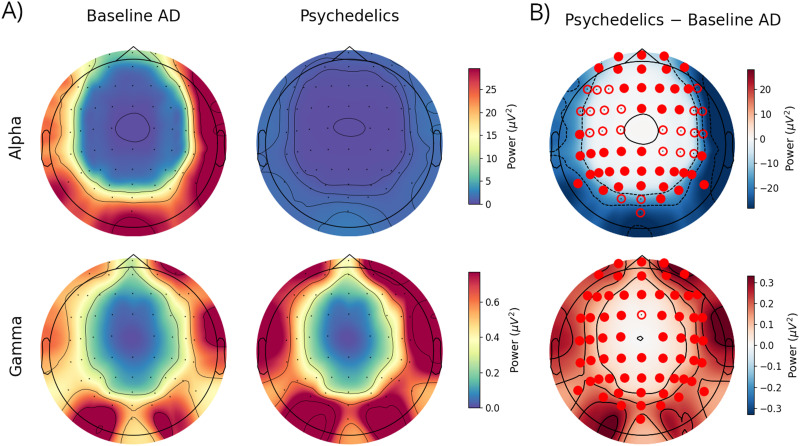
Model reproduces EEG alpha and gamma changes following 5-HT2AR stimulation with psychedelics. (A) Subject-averaged topographic map of the mean alpha and gamma band's EEG PSD for the AD whole-brain models with and without stimulation by psychedelics. Black dots denote the recording location of the EEG electrodes. (B) Subject-averaged EEG PSD difference between the baseline AD models and the models with psychedelics effects (psychedelics − baseline). The statistical significance (Bonferroni-corrected) of the PSD changes in each EEG channel is denoted by an empty red dot (*p* < 0.05) or a filled red dot (*p* < 0.001) on top of the corresponding electrode.

Finally, we studied the power spectrum alterations induced by psychedelics in EEG data. Figure 7A shows the subject-averaged topographic map of the power in the alpha and gamma bands computed from the simulated EEG signals in the AD whole-brain models with and without psychedelics. The subject-averaged PSD difference between the baseline and psychedelic conditions is shown in Figure 7B, along with the statistical significance of the changes in each EEG electrode given by the analysis described above.

Consistent with our previous results, the EEG shows a brain-wide decrease of alpha power in the spontaneous brain activity of all the modeled subjects (62% mean alpha PSD decrease with respect to baseline averaged over subjects and EEG channels). This decrease is significant across all EEG channels, and highly significant decreases in alpha power (*p* < 0.001) were consistently observed in all subjects and in all brain lobes. On the other hand, we found a significant increase in the EEG gamma band power after 5-HT2AR stimulation (37% mean gamma PSD increase with respect to baseline averaged over subjects and EEG channels). This increase is significant across all EEG channels and was consistently observed in all subjects.

It is worth mentioning that the cortical distribution of alpha and gamma activity in the synthetic EEG may not fully align with the spectral features typically observed in the EEG of healthy or AD individuals. The reduced oscillatory activity in both alpha and gamma frequencies in the parietal regions may be explained by the fact that the whole-brain models developed in this study were not personalized based on EEG data but on fMRI data. Inspection of the connectivity matrix revealed that this reduced EEG power can be mostly explained by a saturation of the connections to the parietal lobe. Indeed, the parietal lobe acts as a network hub, and the neural populations in this lobe receive high inputs and consequently often show saturated activity in the model. This hinders the emergence of oscillatory activity in alpha and gamma frequencies, resulting in the decreased alpha and gamma power observed in the parietal and parieto-frontal areas.

### Simulated EEG Complexity and Entropy

**Figure 8.**
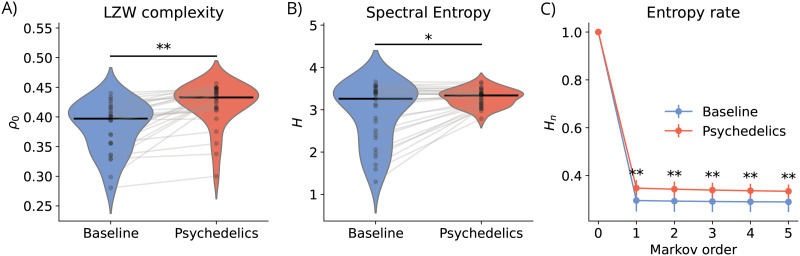
Model reproduces complexity and entropy changes under psychedelics. (A) Normalized LZW complexity of broadband EEG for the whole-brain models at baseline and under psychedelics. A black line indicates the median complexity, and individual dots connected by gray lines represent the signal complexity for each subject. (B) Spectral entropy of broadband EEG signals for both conditions. (C) Entropy rate for different Markov orders of broadband EEG signals in the baseline and psychedelics conditions. Error bars indicate the standard deviation across subjects. In all cases, the significance level of the difference between conditions resulting from a paired *t* test (Bonferroni-corrected for the entropy rate) is denoted by the number of asterisks (*p < 0.001, **p < 0.0001).

Next, we used different metrics to capture the complexity of the simulated EEG signals obtained from the personalized hybrid brain models under psychedelics. Given the nonlinear dynamics of EEG signals, we selected metrics that estimate upper bounds on algorithmic complexity. The normalized LZW complexity (*ρ*_0_) of EEG signals in subjects under psychedelics was significantly higher than at baseline (Figure 8A), demonstrating that the oscillatory changes induced by psychedelics in the whole-brain models lead to increased complexity of the signals.

This was further highlighted by the significant increase in spectral entropy (Figure 8B) and entropy rate at different Markov orders (Figure 8C). The normalized LZW complexity reflects the structural richness of the signal, while the spectral entropy indicates a more uniform power distribution across frequencies, consistent with increased neural variability mainly explained by the decreased power in the alpha band (Figure 5B). The entropy rate at different orders provides a measure of the temporal predictability of the EEG signals, highlighting how psychedelics induce more diverse oscillatory patterns in the models.

## DISCUSSION

### Main Findings

In our model, we implemented psychedelic effects as acting selectively on L5 pyramidal neurons (*P*_1_), consistent with their role as the main cortical targets of 5-HT2A receptors (Nichols, 2016). As a result, alpha oscillations, primarily generated by this population, were directly affected, while gamma changes emerged only indirectly through the interactions between neuronal populations within the LaNMM.

Our modeling implementation of psychedelics' effects into NMMs successfully explains the power spectrum typically observed after administration of psychedelics in the experimental literature (Carhart-Harris & Friston, 2019; Carhart-Harris et al., 2014; Ort et al., 2023; Timmermann et al., 2023). Indeed, the oscillatory changes found in our personalized whole-brain models following implementation of 5-HT2AR activation closely align with the findings of Timmermann et al. (2023), where a significant decrease in mean alpha and beta band power, together with a significant increase in mean gamma power, was found in the acute phase of DMT injection in healthy subjects. Ort et al. (2023) also reported similar results, showing that the mean theta and alpha powers were significantly decreased in spontaneous EEG PSD under psilocybin, although no significant changes were observed in other frequencies such as the gamma band.

We found a strong correlation between the changes in mean alpha and gamma power across the brain and the density of 5-HT2ARs in each brain area. Consequently, and consistent with our initial hypothesis, the significantly reduced alpha power and increased gamma power observed under the influence of psychedelics are mainly explained in our modeling framework by the agonism of these drugs on 5-HT2A receptors and the magnitude and spatial distribution of their effects are directly related to the expression amounts of such serotonin receptors.

Our findings align with experimental evidence linking psychedelics to enhanced neural entropy and complexity (Carhart-Harris & Friston, 2019; Carhart-Harris et al., 2014; Ruffini et al., 2023), and they support the potential therapeutic role of psychedelics in restoring the complexity deficits observed in AD (Abásolo et al., 2006; Sun et al., 2020). Different metrics of complexity and entropy (LZW, spectral entropy, and entropy rate) resulted in increased signal diversity and richness in the personalized models under psychedelics with respect to baseline. The ability of our whole-brain models to reproduce these effects highlights their utility in exploring the neural mechanisms underlying psychedelic-induced changes and their potential for counteracting pathological states associated with reduced complexity.

### Potential Therapeutic Role of Psychedelics in AD

Our results suggest that psychedelics may acutely mitigate EEG spectral alterations characteristic of AD, reinforcing their therapeutic potential for this neurodegenerative condition. The spectral shifts induced by psychedelics in our models may counteract the disrupted alpha–gamma coupling and oscillatory imbalances observed in AD induced by PV dysfunction, potentially improving FC and cognition. These results indicate that psychedelics may hold therapeutic potential in the AD stages characterized by alpha power increases and reduced gamma power (prodromal and mild AD). Consistent with this interpretation, analysis of the simulated activity of the PV population revealed a small but significant increase in both firing rate and gamma power under psychedelics (see Supporting Information S9), suggesting an enhancement of PV-mediated inhibitory dynamics.

However, we caution that in the preclinical and very early stages of AD, cortical neurons in certain regions exhibit heightened excitability, driven partly by elevated concentrations of soluble amyloid-beta, which impairs glutamate uptake and increases extracellular glutamate levels (Bao et al., 2021; Harris, Wolf, De Strooper, & Busche, 2020; Zott et al., 2019). As activation of 5-HT2ARs by psychedelics modulates glutamate's excitatory effects, increasing the excitability of deep-layer pyramidal neurons (Andrade, 2011), their use under these conditions may intensify the existing hyperexcitability and accelerate pathological processes, suggesting a contraindication of such compounds during the earliest phases of AD. On the other hand, in the later stages of AD, the widespread loss of alpha and gamma power resulting from global neuronal death may render psychedelics ineffective as a treatment. This suggests there may be an optimal window for the treatment of AD with psychedelics, namely, the prodromal to mild phase.

Importantly, the effects of psychedelics are not limited to their acute phase. Growing evidence highlights a postacute phase characterized by enhanced neural plasticity, which underpins their long-term therapeutic effects (Calder & Hasler, 2023; Ruffini, Lopez-Sola, Vohryzek, & Sanchez-Todo, 2024). This phase involves structural and functional neural changes that can promote sustained recovery of cognitive and neural functions, which is particularly relevant for addressing the progressive degeneration seen in AD. Thus, by recalibrating neuronal excitability and oscillatory activity, psychedelics may not only acutely alleviate acute EEG abnormalities but also facilitate long-term neural reorganization and plasticity. This aligns with prior research emphasizing the role of enhanced brain FC and neuroplasticity in slowing or reversing neurodegeneration (Garcia-Romeu et al., 2022; Winkelman et al., 2023). In the present work, we focused exclusively on the acute electrophysiological effects of psychedelics, and modeling their long-term impact on neural plasticity and network reorganization remains an important avenue for future research.

Finally, there is a natural synergy between psychedelics and noninvasive interventions such as transcranial electrical stimulation (Dhaynaut et al., 2022; Sprugnoli et al., 2021) or sensory stimulation (Adaikkan et al., 2019; Adaikkan & Tsai, 2020; Iaccarino et al., 2016; Martorell et al., 2019), which seek to entrain natural fast frequency circuits in AD. The combination of psychedelics with such interventions can produce benefits from the acute effects of each and by the increased window of plasticity facilitated by psychedelics (Ruffini, Castaldo, Lopez-Sola, Sanchez-Todo, & Vohryzek, 2024; Ruffini, Lopez-Sola, et al., 2024).

### Limitations and Future Work

Several limitations in the present study are worth pointing out. Although our focus is on AD, the inclusion of data from healthy subjects could offer a critical comparative framework. By contrasting the altered alpha and gamma power in AD models treated with psychedelics against the normative power spectrum dynamics observed in healthy individuals, we might better understand the restorative potential of psychedelics. Such comparisons could elucidate whether the modeled oscillatory dynamics approximate the canonical signatures of healthy brain activity.

Another consideration concerns the characterization of spectral features. While PSD estimates may be unreliable for short or single-trajectory data (Krapf et al., 2019), in our study, PSDs were computed from long time series across 84 cortical parcels, providing robust and well-sampled estimates. Nevertheless, complementary scale-free approaches such as the Hurst exponent (Hardstone et al., 2012) could further enrich the analysis and represent an interesting avenue for future work.

At the modeling level, our AD model represents pathology only at the level of global connectivity and external noise. In parallel work in mesoscale modeling of AD, we have developed an adapted version of the LaNMM that reproduces the impact of PV synapse damage in oscillatory dynamics, producing slowing of EEG, increased alpha power, and reduced gamma (Sanchez-Todo et al., 2024). In future work, we can investigate the effects of psychedelics in this AD mesoscale model, which incorporates physiologically inspired mechanisms to reproduce the disruptions in oscillatory activity observed in AD. Moreover, our models were fitted using fMRI-derived FC, but we did not explore simulated BOLD-level changes under psychedelics. Future work could examine whether the models reproduce reported fMRI signatures of psychedelics, such as increased global integration and reduced modularity, although these effects may differ in the context of AD.

Finally, empirical validation remains a key next step. Combined EEG–fMRI studies in both healthy individuals and early-stage AD patients would enable direct testing of the model's predictions linking receptor density, oscillatory modulation, and large-scale network dynamics. In particular, simultaneous assessment of alpha and gamma power changes alongside alterations in FC could provide crucial evidence for the mechanistic hypotheses proposed here.

## CONCLUSIONS

In this study, we explored the mechanisms behind spectral power alterations during the acute phase of serotonergic psychedelic drug effects and provided a novel mechanistic understanding of their impact on brain oscillatory dynamics. By implementing 5-HT2A receptor activation in an LaNMM, we reproduced the characteristic EEG multiband power spectrum alterations observed under psychedelics within a single cortical column. Extending this approach, we used 30 subject-specific whole-brain models, integrating MRI, dMRI, and fMRI data from AD subjects, to simulate the effects of psychedelics on whole-brain dynamics following 5-HT2A receptor stimulation.

The subject-specific whole-brain models effectively reproduced the power spectrum changes observed experimentally with psychedelics, notably the significant decreases in alpha and beta powers and increases in gamma power. Our findings align with previous studies combining fMRI and EEG approaches to psychedelics (Ort et al., 2023; Timmermann et al., 2023) and further demonstrate a strong correlation between modulation of mean alpha and gamma power across brain regions and the spatial distribution of 5-HT2A receptors. By combining these whole-brain models with a biophysical head model, we successfully reproduced the suppression of alpha rhythms and increased gamma power observed in prior EEG studies as well as the complexity changes associated with the effects of psychedelics.

This modeling framework offers new insights into the neural mechanisms underlying psychedelic action and highlights the potential of psychedelics to restore healthy oscillatory dynamics in disorders like AD. Our approach paves the way for using computational whole-brain models to investigate the potential of psychedelics in rebalancing neural activity in neurodegenerative and psychiatric disorders.

## Acknowledgments

We want to thank the Elite Master Program in Neuroengineering at the Technical University of Munich, funded by the Elite Network of Bavaria, for the financial support that facilitated the publication of this work.

## Supporting Information

Supporting information for this article is available at https://doi.org/10.1162/NETN.a.540.

## Author Contributions

Jan C. Gendra: Conceptualization; Formal analysis; Funding acquisition; Investigation; Methodology; Software; Visualization; Writing – original draft; Writing – review & editing. Edmundo Lopez-Sola: Conceptualization; Formal analysis; Methodology; Software; Visualization; Writing – original draft; Writing – review & editing. Francesca Castaldo: Conceptualization; Writing – review & editing. Èlia Lleal-Custey: Methodology; Software; Writing – review & editing. Roser Sanchez-Todo: Conceptualization; Visualization; Writing – review & editing. Jakub Vohryzek: Conceptualization; Writing – review & editing. Ricardo Salvador: Methodology; Software; Writing – review & editing. Ralph G. Andrzejak: Conceptualization; Writing – review & editing. Giulio Ruffini: Conceptualization; Funding acquisition; Methodology; Software; Writing – review & editing.

## Competing Interests

Competing Interests: Edmundo Lopez-Sola, Francesca Castaldo, Ricardo Salvador, Elia Lleal, Roser Sanchez-Todo, and Giulio Ruffini work for Neuroelectrics, a company developing computational brain stimulation solutions for neuropsychiatric disorders.

## Funding Information

Edmundo Lopez-Sola, H2020 European Research Council (https://dx.doi.org/10.13039/100010663), Award ID: 855109. Francesca Castaldo, H2020 European Research Council (https://dx.doi.org/10.13039/100010663), Award ID: 855109. Èlia Lleal-Custey, H2020 European Research Council (https://dx.doi.org/10.13039/100010663), Award ID: 855109. Roser Sanchez-Todo, H2020 European Research Council (https://dx.doi.org/10.13039/100010663), Award ID: 855109. Ricardo Salvador, H2020 European Research Council (https://dx.doi.org/10.13039/100010663), Award ID: 855109. Giulio Ruffini, H2020 European Research Council (https://dx.doi.org/10.13039/100010663), Award ID: 855109. Edmundo Lopez-Sola, H2020 Future and Emerging Technologies (https://dx.doi.org/10.13039/100010664), Award ID: 101017716. Francesca Castaldo, H2020 Future and Emerging Technologies (https://dx.doi.org/10.13039/100010664), Award ID: 101017716. Èlia Lleal-Custey, H2020 Future and Emerging Technologies (https://dx.doi.org/10.13039/100010664), Award ID: 101017716. Roser Sanchez-Todo, H2020 Future and Emerging Technologies (https://dx.doi.org/10.13039/100010664), Award ID: 101017716. Jakub Vohryzek, H2020 Future and Emerging Technologies (https://dx.doi.org/10.13039/100010664), Award ID: 101017716. Ricardo Salvador, H2020 Future and Emerging Technologies (https://dx.doi.org/10.13039/100010664), Award ID: 101017716. Giulio Ruffini, H2020 Future and Emerging Technologies (https://dx.doi.org/10.13039/100010664), Award ID: 101017716. Ralph G. Andrzejak, Agencia Estatal de Investigación (https://dx.doi.org/10.13039/501100011033), Award ID: PID2020118196GBI00/MICIU/AEI/10.13039/501100011033. Jan C. Gendra, Elitenetzwerk Bayern (https://dx.doi.org/10.13039/501100008848).

## Data Availability

Data used in the preparation of this article were obtained from the ADNI database (https://adni.loni.usc.edu). The ADNI is a longitudinal, multicenter study aimed at developing clinical, imaging, genetic, and biochemical biomarkers for the early detection and tracking of AD. A key feature of ADNI is its open data-sharing policy, which allows qualified researchers worldwide to access a comprehensive collection of de-identified data, including structural, functional, and molecular brain imaging, biofluid biomarkers, cognitive assessments, genetic data, and demographic information.

## Supplementary Material



## TECHNICAL TERMS

Serotonin 2A receptor (5-HT2AR): Cortical serotonin receptor strongly expressed on pyramidal neurons that modulates excitability and drives key psychedelic neural effects.
Power spectral density (PSD): Quantifies how signal power distributes across frequencies, revealing dominant oscillatory rhythms.
Neural mass model (NMM): Mathematical framework describing the collective activity of neuronal populations using averaged firing and synaptic dynamics.
Laminar neural mass model (LaNMM): Extension of NMMs that incorporates cortical layers to simulate interacting alpha- and gamma-generating circuits.
Hybrid brain model: Model combining a neurophysiological whole-brain model with a biophysical head model.
Diffusion MRI (dMRI): Imaging method mapping white matter pathways by modeling directional water diffusion along axonal fibers.
Personalized whole-brain model: Computational model whose parameters are tuned to individual anatomical and functional data to reproduce subject-specific dynamics.
Resting-state fMRI (rs-fMRI): fMRI recorded without tasks to measure spontaneous large-scale brain network activity.
Structural connectome (SC): Network of anatomical connections between brain regions reconstructed from diffusion MRI tractography.
Functional connectivity (FC): Statistical relationship between activity time series in different brain regions, indexing coordinated neural dynamics.
Balloon–Windkessel model: Biophysical model translating neuronal activity into predicted BOLD responses using vascular dynamics and blood volume changes.
BOLD signal: fMRI signal reflecting blood oxygenation changes that indirectly track population-level neural activity.
