# Supplementary material for: Restoring oscillatory dynamics in Alzheimer's disease: A laminar whole-brain model of serotonergic psychedelic effects
Source: Netw Neurosci. 2026 Apr 22;10(2):303–28. doi: 10.1162/NETN.a.540 (PMC13108508; doi:10.1162/NETN.a.540)
Supplement: Supplementary file 1 [file netn-10-2-303-s001.pdf]

Supporting Information

Restoring Oscillatory Dynamics in Alzheimer's Disease: A Laminar Whole-Brain Model of Serotonergic Psychedelic Effects

Jan C. Gendra[1,2,3,*], Edmundo Lopez-Sola[2,4,*], Francesca Castaldo[2], Èlia Lleal-Custey[2], Roser Sanchez-Todo[2,4], Jakub Vohryzek[4], Ricardo Salvador[2], Ralph G. Andrzejak[3], and Giulio Ruffini[2], for the Alzheimer's Disease Neuroimaging Initiative[†]

[1]School of Computation, Information and Technology, Technical University of Munich, Munich, Germany

[2]Brain Modeling Department, Neuroelectrics, Barcelona, Spain

[3]Department of Engineering, Universitat Pompeu Fabra, Barcelona, Spain

[4]Center for Brain and Cognition, Universitat Pompeu Fabra, Barcelona, Spain

[*]Equal contribution

[†]Data used in the preparation of this article were obtained from the Alzheimer's Disease Neuroimaging Initiative (ADNI) database (http://adni.loni.usc.edu). As such, the investigators within the ADNI contributed to the design and implementation of ADNI and/or provided data but did not participate in the analysis or writing of this report. A complete listing of ADNI investigators can be found at: http://adni.loni.usc.edu/wp-content/uploads/how_to_apply/ADNI_Acknowledgement_List.pdf

S1 AD STAGES AND PHYSIOPATHOLOGY

Stages of AD and electrophysiological alterations

AD progresses through distinct stages, beginning with a *preclinical* phase, characterized by pathological changes such as amyloid-beta and tau accumulation detectable through biomarkers, but without noticeable cognitive symptoms (Sperling et al., 2011). The next stage, *prodromal AD* or Mild Cognitive Impairment (MCI) due to AD, involves subtle cognitive declines, particularly in memory, that exceed normal aging but do not yet significantly impair daily life (Petersen, 2004). The disease progresses then from *mild AD dementia*, where cognitive impairments interfere with daily tasks, to *moderate AD dementia* with severe cognitive decline and increased dependency, and ultimately to *severe AD dementia* marked by extensive neurodegeneration (Alzheimer's, 2020).

In the *preclinical stages of AD*, some studies report an increase in alpha power along with a potential increase in gamma power (Gaubert et al., 2019; Nakamura et al., 2018). However, findings regarding gamma power in this stage are variable, with some evidence suggesting stable or mildly reduced gamma activity (Rochart, Liu, Fonteh, Harrington, & Arakaki, 2020). After the preclinical stage of AD, the EEG spectrum undergoes a characteristic slowing, with increased power in lower frequencies (delta and theta) and decreased power in higher frequencies (beta and gamma) as the disease progresses (Babiloni et al., 2020; Casula et al., 2022; Murty et al., 2021). Increased alpha power and hypersynchronization in alpha-band functional connectivity reflect an imbalance in excitation and inhibition, which contributes to neuronal hyperactivity and network disruptions (López et al., 2014; Nakamura et al., 2018), although there is also evidence of decreased alpha power in MCI patients (López-Sanz et al., 2016). As the disease advances into *early to moderate AD*, alpha power begins to decline, and a slowing of the alpha peak frequency is observed, which correlates with structural and functional impairments (Garcés et al., 2013; Passero, Rocchi, Vatti, Burgalassi, & Battistini, 1995; Puttaert et al., 2021). Gamma oscillations, which are essential for higher-order cognitive functions like working memory and sensory processing, show a more consistent decrease during MCI and early AD, reflecting synaptic dysfunction and impaired connectivity (Babiloni et al., 2020; Murty et al., 2021; Sanchez-Todo, Lopez-Sola, Mercadal, & Ruffini, 2024). In *later stages of AD*, both alpha and gamma power are markedly reduced, alongside significant increases in slower rhythms such

as delta and theta. This shift in the EEG power spectrum mirrors widespread cortical atrophy, disrupted excitatory-inhibitory balance, and breakdowns in network synchronization and efficiency, which underpin the severity of cognitive and functional decline (Babiloni et al., 2020; Casula et al., 2022).

Physiopathology of AD

AD is associated with the dysfunction of inhibitory interneurons, particularly those expressing parvalbumin (PV), and the resulting network abnormalities in AD (Palop & Mucke, 2016; Sanchez-Todo et al., 2024). PV interneurons, crucial for maintaining fast oscillations, become impaired in AD due to both direct pathology and, possibly, compensatory neural changes (Palop & Mucke, 2016). This dysfunction leads to a loss of gamma rhythm, essential for higher cognitive processes, and contributes to network instability and hypersynchrony in slower frequencies.

In early AD, synaptic connections from PV interneurons in superficial layers, critical for generating fast gamma rhythms, are among the first to be disrupted (Palop & Mucke, 2016). This early damage corresponds with the emergence of increased alpha and reduced gamma activity observed in MCI and mild-to-moderate AD (López-Sanz et al., 2017). Eventually, persistent alpha hypersynchronization further deteriorates these circuits and affects other connected areas in later disease stages. The precise mechanisms linking PV interneuron dysfunction to EEG slowing remain unclear, and compensatory processes have been suggested (Gaubert et al., 2019).

S2 LANMM DESCRIPTION

S2.1 Single LaNMM parameters

The list of LaNMM parameters used for the simulations is shown in Table S1. Model parameters were adopted from Sanchez-Todo and Bastos (2023), except for the standard deviation σ_e of the external noisy perturbations e_1 and e_2. In the single LaNMM study, we adjusted σ_e to match the average SD value of the external inputs received by each LaNMM in the whole-brain model personalization (see Section S2.3).

Table S1: Parameters, description, and values of the baseline Laminar NMM employed in this work. The values are taken from Sanchez-Todo and Bastos (2023), except for the external inputs (φ_{e1}, φ_{e2}). Notice that the sigmoid function parameters defining the firing rate φ_m in this model (ν_0, φ_0, r), apply to all neuron populations. Moreover, all excitatory connections follow the same synapse dynamics, $(A, a)_{\mathrm{AMPA}} = (A, a)_{1,3,4,5,6,8,9,11,12,13}$ while all inhibitory connections follow either fast, $(A, a)_{\mathrm{GABA_{fast}}} = (A, a)_{7,10}$, or slow dynamics, $(A, a)_{\mathrm{GABA_{slow}}} = (A, a)_2$.

Parameter	Description	Value
A_s	Average excitatory and (fast and slow) inhibitory synaptic gains	$A_{\mathrm{AMPA}} = 3.25$ mV<br>$A_{\mathrm{GABA_{fast}}} = -22$ mV<br>$A_{\mathrm{GABA_{slow}}} = -30$ mV
a_s	Time constant of average excitatory and inhibitory postsynaptic potentials	$a_{\mathrm{AMPA}} = 100$ Hz<br>$a_{\mathrm{GABA_{fast}}} = 50$ Hz<br>$a_{\mathrm{GABA_{slow}}} = 220$ Hz
C_s	Connectivity constant between populations	$C_1 = 108,\ C_2 = 33.75$<br>$C_3 = 1,\ C_4 = 135$<br>$C_5 = 33.75,\ C_6 = 70$<br>$C_7 = 550,\ C_8 = 1$<br>$C_9 = 200,\ C_{10} = 100$<br>$C_{11} = 80,\ C_{12} = 200$<br>$C_{13} = 30$
ν_0	Membrane potential at 50% of the firing rate	6 mV<br>(except P_2: $\nu_0 = 1$ mV)
φ_0	Half of the maximum firing rate	2.5 Hz
r	Slope of the sigmoid function at v_0	0.56 mV^{-1}
μ_e, σ_e	Mean and SD of the external input firing rates φ_e	φ_{e1}: white noise with<br>$\mu_{e1} = 270$ Hz and $\sigma_{e1} = 34$ Hz<br>φ_{e2}: white noise with<br>$\mu_{e2} = 90$ Hz and $\sigma_{e2} = 34$ Hz

S2.2 Long-range LaNMM connectivity

The long-range connections between nodes in the model are weighted following the connectivity patterns between cortical regions described by Bastos et al. (2012) and recent modeling work (Jaramillo, Mejias, & Wang, 2019; Mejias, Murray, Kennedy, & Wang, 2016). In particular, P_1 and P_2 populations of different parcels i, j are connected as follows: $P_1^i \to P_1^j$ (lateral connection), $P_1^i \to P_2^j$ (feedback or descending connection) and $P_2^i \to P_2^j$ (feedforward or ascending connection) with relative weights w of 0.5, 0.5 and 1 respectively (see Figure S1B). The resulting connectivity C_{ij} between parcels i and j depends on which of these three connections is involved, and is computed as:

$$C_{ij} = w \cdot G \cdot K_{ij} \tag{S1}$$

w represents the relative strength of the connection, G is the global scaling factor that adjusts the overall connection strength and K_{ij} is the connectome value. For simplicity, we assumed that all long-range cortico-cortical connections across brain columns are mediated via pyramidal populations — hence, they all have an excitatory effect.

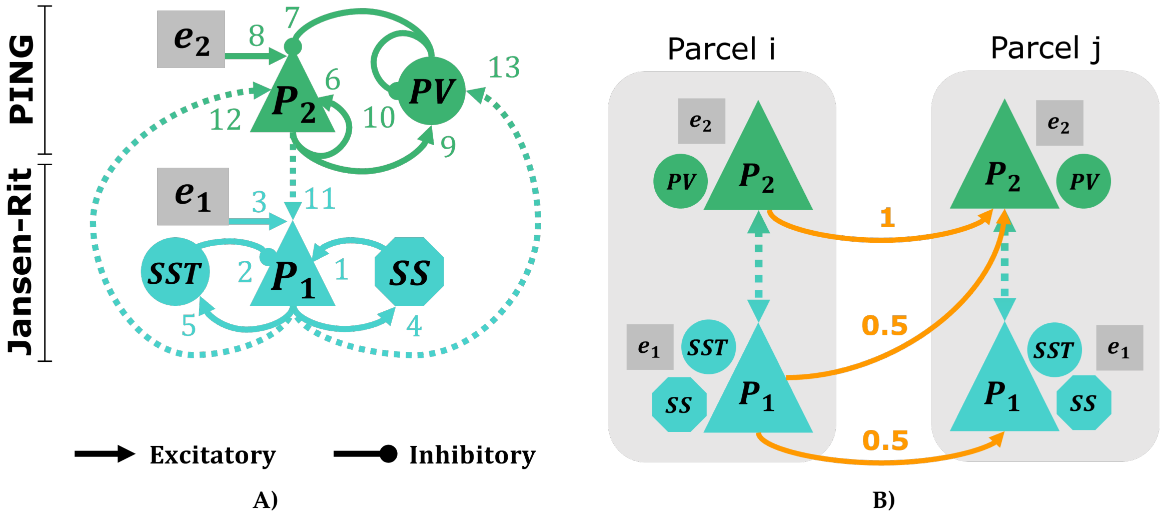

Figure S1: (A) Schematic diagram of the LaNMM depicting its neuronal populations and the connections between them. The PING sub-circuit is depicted in green and the JR sub-circuit in blue. Rounded shapes indicate inhibitory populations and interactions, triangles and octagons indicate excitatory ones, and squares represent external inputs. Numbers indicate the index s assigned to each connection. Dashed lines represent connections between the PING and Jansen-Rit parts sub-circuits. The neural population abbreviations (P_1, SS, SST, P_2, PV, $e1$, $e2$) are defined in the main text. **(B)** Long-range cortico-cortical parcel connections and their relative strength. Note that, for simplicity, cortico-cortical connections are only drawn in one direction, but they are present in both directions in the model.

S2.3 Whole-brain model external inputs

The external noisy inputs $e1$ and $e2$ applied to the JR and PING model's pyramidal populations (as described in Methods, Section *Laminar neural mass model implementation* of the main text) are modeled in the whole-brain models as the sum of two additive noises:

- On the one hand, a *common noise* is applied homogeneously to all parcels (i.e., the same realization of noise is applied to every parcel) and modeled as white Gaussian noise with mean μ_{c1} = 270 Hz to P_1 and mean μ_{c2} = 90 Hz to P_2, mirroring the values of the single LaNMM (Sanchez-Todo & Bastos, 2023). The SD of the common noise σ_c is personalized for every subject.
- On top of this, white Gaussian noise with mean μ_i = 0 Hz and SD σ_i = 20 Hz is added to the common noise in both P_1 and P_2 *homotopically*, i.e., the same realization of noise is applied to "mirror" areas of homologous parcels in both hemispheres—e.g., same noise realization to the left and right insula.

These two external inputs model the influence of FC pathways involving subcortical structures such as the thalamus (Uddin et al., 2008; Wang, Leong, Chan, Liu, & Wu, 2019), constituting a rough thalamo-cortical model, which may play a pivotal role in the context of psychedelics (Müller et al., 2017).

S3 NEUROIMAGING DATA ACQUISITION PARAMETERS

The model personalization pipeline involves three types of neuroimaging data: MRI, dMRI, and blood-oxigen-level-dependent (BOLD) resting-state fMRI (rs-fMRI). The data acquisition parameters for these modalities and the patient cohort selected is provided below.

T1-weighted MRI images were acquired using standardized MPRAGE protocols with 3T MRI scanners across multiple sites. Key acquisition parameters included TR = 2300 ms, TE = 2.98 ms, voxel size = 1×1×1 mm, and flip angle = 9°. Resting-state fMRI data were acquired using standardized ADNI3 protocols with gradient-echo EPI sequences. Key parameters included TR = 3000 ms, TE = 30 ms, flip angle = 90°, voxel size = 3.3×3.3×3.3 mm, and scan duration = ∼6 minutes. Diffusion MRI was acquired with b-values of 0 and 1000 s/mm^2, 41 gradient directions, TR = ∼9100 ms, TE = ∼98 ms, and voxel size = 2.7×2.7×2.7 mm. Detailed imaging protocols and scanner-specific adjustments are available in the ADNI3 documentation.

S4 PARCELLATION OF PET-DERIVED 5-HT2AR DENSITY IN DK-68

To obtain a quantitative measure of the 5-HT2AR density in each DK-68 parcel, we parcellated Beliveau et al.'s 5-HT2AR average density map into the DK-68 cortical atlas. For this purpose, we used the *Neuromaps* toolbox (Markello et al., 2022) and followed Hansen et al.'s parcellation pipeline (Hansen et al., 2022). We used the DK-68 average subject template atlas automatically labeled by FreeSurfer 7.4 in *fsaverage* space (Fischl, 2012; Fischl et al., 2004) to obtain the average 5-HT2AR density in the surface area of each cortical parcel. The PET imaging data used in this study, including receptor images and densities, are included in Neuromaps[1] and the DK-68 *fsaverage* atlas is available in the installation files of *FreeSurfer* 7.4[2]. The obtained receptor map and the DK-68 parcellation used can be found in Figure S2.

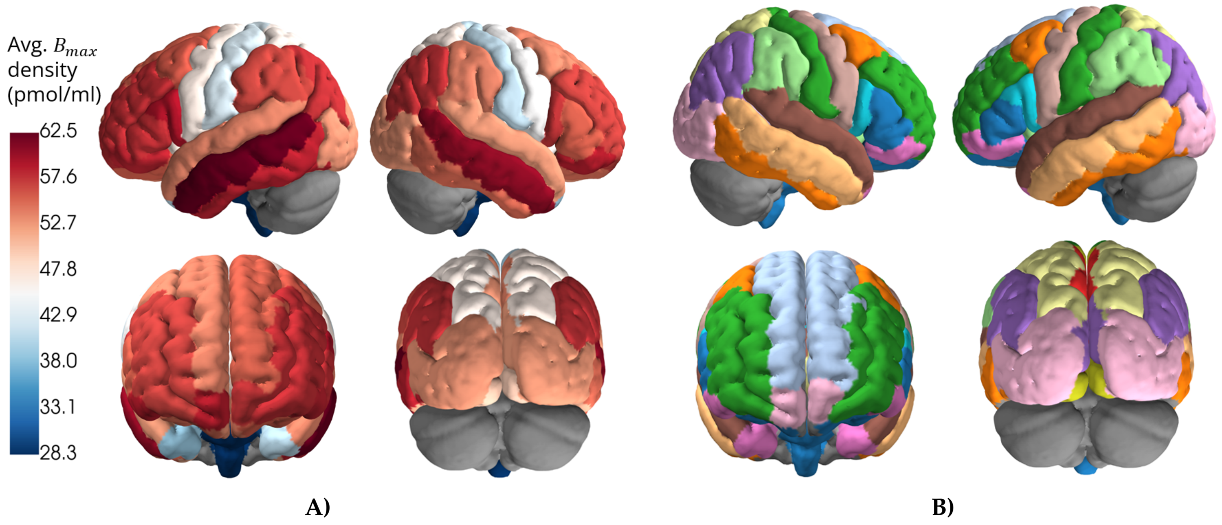

Figure S2: (A) 5-HT2A receptor map of average B_{max} densities parcellated into the DK-68 atlas. **(B)** DK-68 atlas displaying each parcel in distinct colors, with homotopic parcels sharing the same color. Excluded areas (i.e., subcortical structures) are displayed in grey.

[1] https://github.com/netneurolab/neuromaps
[2] http://surfer.nmr.mgh.harvard.edu

S5 BIOPHYSICAL HEAD MODEL GENERATION

Figure S3 illustrates the process of creating the template head model. The head model was generated from a template T1-weighted image (MNI-ICBM 152 non-linear 2009[3]). Segmentation was performed with a pre-trained convolutional neural network (based on a stacked 2D U-Net (Ronneberger, Fischer, & Brox, 2015)), generating volume masks for the scalp, skull, CSF (including the ventricles), GM and WM. All segmentation masks were manually inspected and corrected as needed. The volume segmentations were then converted to triangulated surface meshes and then to a tetrahedral volume mesh using the SimNIBS library v3.2.3[4].

The lead field matrix was then calculated for all electrode positions defined in the 10-10 EEG positioning system (Jurcak, Tsuzuki, & Dan, 2007). This is a $N_{\text{mesh nodes}} \times N_{\text{electrodes}} - 1$ matrix where each column contains the E-field component orthogonal (E_n) to the cortical surface (GM-CSF interface) induced by a bipolar combination of electrodes where Cz is always defined as the cathode (-1 mA). Electrodes were modeled as a cylinder of gel with a height of 3 mm and a radius of 1 cm (representing Neuroelectrics' NG-Pistims[5]). E-field calculations were performed with the SimNIBS library, assigning to each head tissue an isotropic and homogeneous conductivity: 0.33 S/m, 0.008 S/m, 1.79 S/m, 0.4 S/m and 0.15 S/m, respectively for scalp, skull, CSF (including the ventricles), GM and WM. The gel representing the electrodes was modeled with a conductivity of 4.0 S/m.

The lead-field matrix was then used to map activity from source space to electrodes using the reciprocity theorem as described in Ruffini (2015). This was performed by first mapping source activity per parcel to activity per mesh node. To do so, initially the activity of a parcel was assigned to all the mesh nodes in the parcel. This initial map was then blurred by applying a linear operator (**B**) that changes the activity per node to a weighted average of the activity of neighboring nodes, where the weights are determined based on the geodesic distance between nodes and the area of each node of the mesh (defined as the sum of the areas of all the triangles of the mesh that share the node, divided by 3). The electrode voltage differences were then calculated by performing the matrix multiplication of the lead-field matrix and the cortical map of the sources.

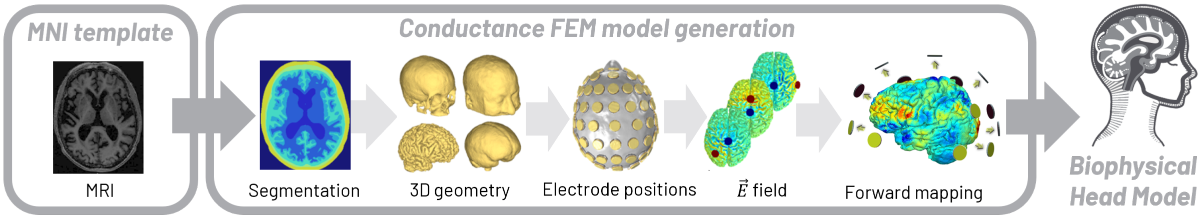

Figure S3: Head model template and cortical mapper generation pipeline. Figure adapted from Salvador et al. (2022)

[3] https://nist.mni.mcgill.ca/atlases/
[4] https://github.com/simnibs/simnibs
[5] https://www.neuroelectrics.com/solution/spareparts-consumables/ngpistim

S6 UNCOUPLED LANMM OSCILLATORY BEHAVIOR

We studied the frequency with the highest PSD (i.e., dominant frequency) in the pyramidal populations over $A_{L5P} \in [0, 10]$ mV, as well as the changes in the membrane potential of P_1 and P_2 resulting from changing the A_{L5P} parameter (Figure S4).

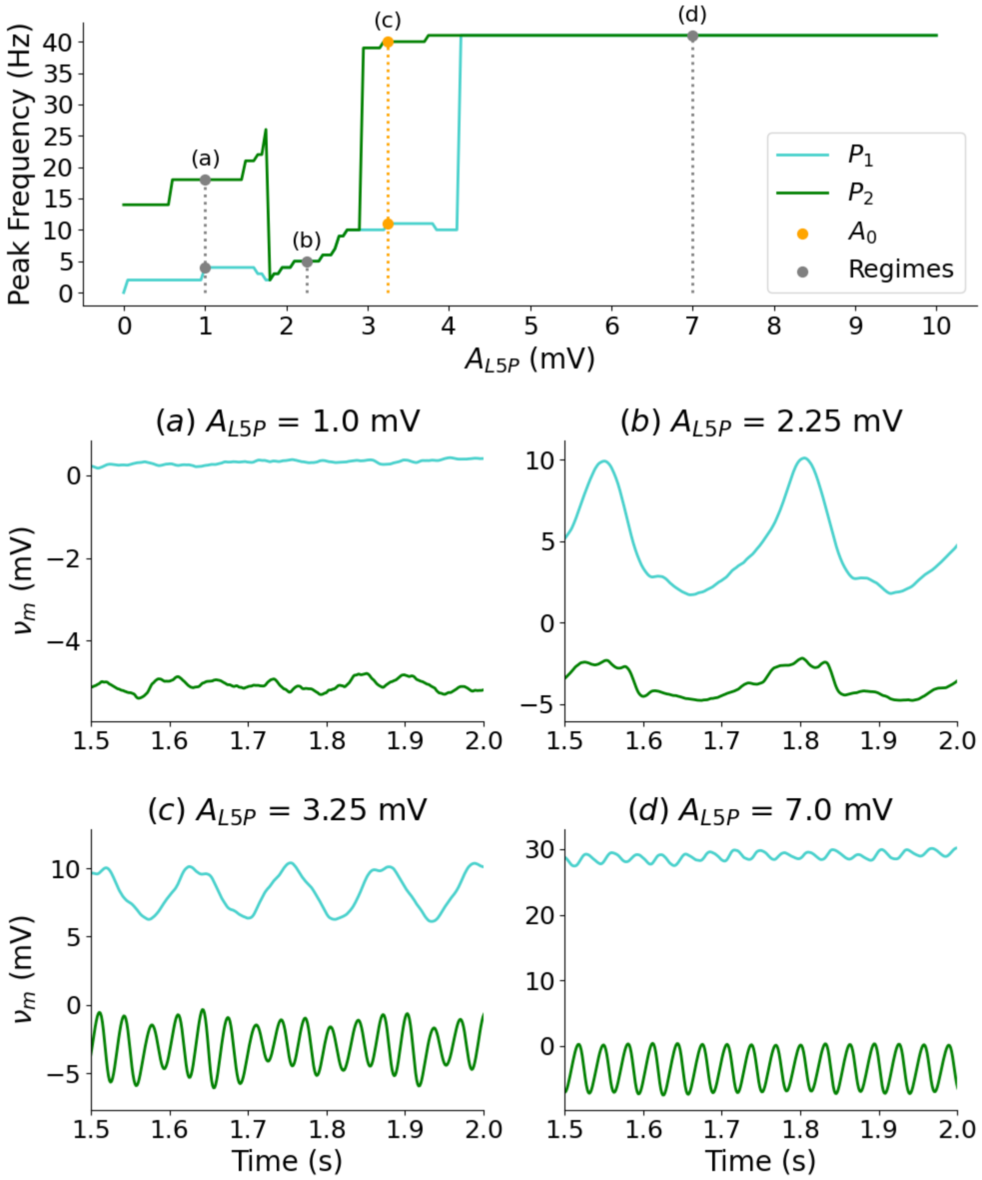

Figure S4: Top: Dominant frequency of the membrane potentials of P_1 (blue) and P_2 (green) pyramidal populations in an uncoupled LaNMM for different values of the synaptic gain A_{L5P} (averaged across five realizations). The orange dashed line denotes the pyramidal gain at baseline A_0 and the grey points denote different model regimes. **Bottom:** Membrane potential of P_1 (blue) and P_2 (green) populations for different A_{L5P} values. The plotted A_{L5P} value is shown on top of each figure. **(A)** No oscillations regime. **(B)** Theta/delta oscillations regime. **(C)** Alpha and gamma oscillations regime. **(D)** Gamma oscillations regime.

We identify four different dynamical regimes in Figure S4:

1. For $A_{L5P} \in [0, 1.8)$ mV (Figure S4A), no oscillations are present in any pyramidal population (noisy regime).
2. For $A_{L5P} \in [1.8, 2.75)$ mV (Figure S4B), theta and delta oscillations are dominant in both pyramidal populations.
3. For $A_{L5P} \in [2.75, 4.15)$ mV (Figure S4C), alpha oscillations are dominant in P_1 and gamma oscillations in P_2 (LaNMM's default regime).
4. For $A_{L5P} \in [4.15, \infty)$ mV (Figure S4D), gamma oscillations are dominant in both pyramidal populations.

To assess whether the spectral power alterations in our model are frequency-specific, we studied the power spectrum of the pyramidal populations over the synaptic gain $A_{L5P} \in [0, 10]$ mV and applied a square window for frequencies $f = [0, 100]$ Hz (Figure S5). The findings show that the area with the highest PSD in P_1 aligns with the delta-theta band for $A_{L5P} \in [2, 2.75]$ mV, the alpha band for $A_{L5P} \in [2.75, 3.75]$ mV, and gamma band for $A_{L5P} > 3.75$ mV. In population P_2, this area lies within the gamma band and agrees with gain values related to gamma oscillations in the previous section. There is also increased PSD in the beta band for both populations, associated with the lower-power harmonics of the alpha band. We conclude that the most significant changes induced by psychedelics in the PSD magnitude in our LaNMM are specific to the alpha and gamma bands within the gain range under study $[A_0, A_{\max}]$ in both P_1 and P_2 populations.

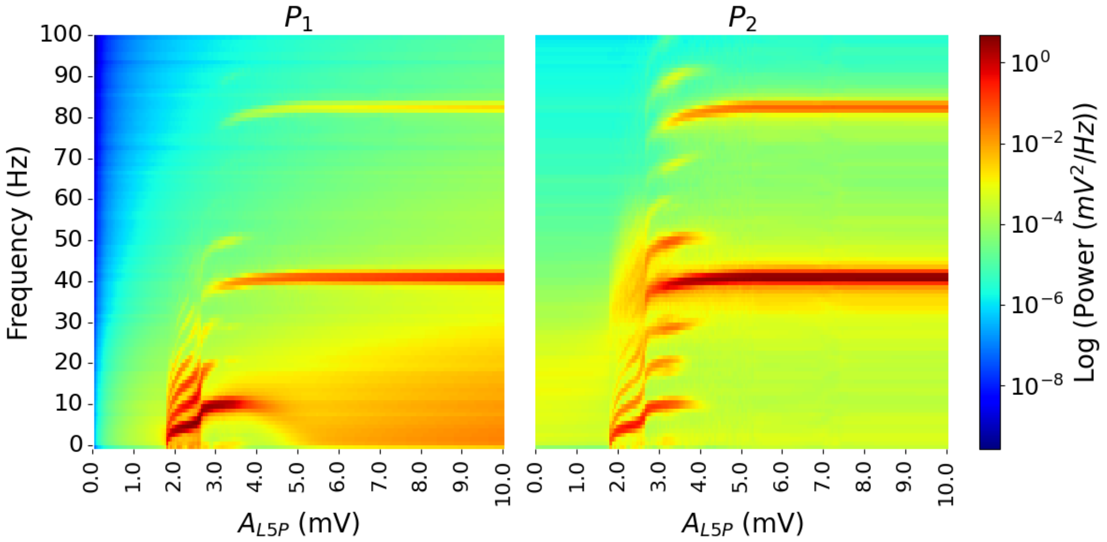

Figure S5: Power spectrum of P_1 (left) and P_2 (right) populations membrane potential over A_{L5P} parameter. The PSD (color scale) is shown in a logarithmic scale.

S7 WHOLE-BRAIN MODEL FITTING RESULTS

Figure S6 and Figure S7 show the values of the optimized parameters G and σ_c/σ_i for each subject after model personalization.

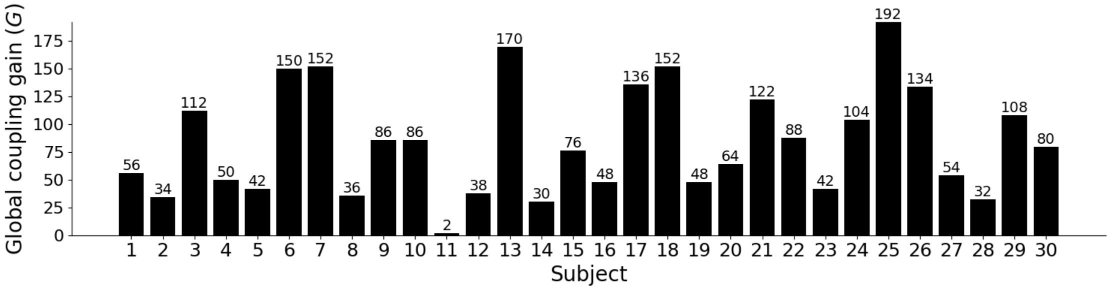

Figure S6: Personalized values of the G parameter after model personalization

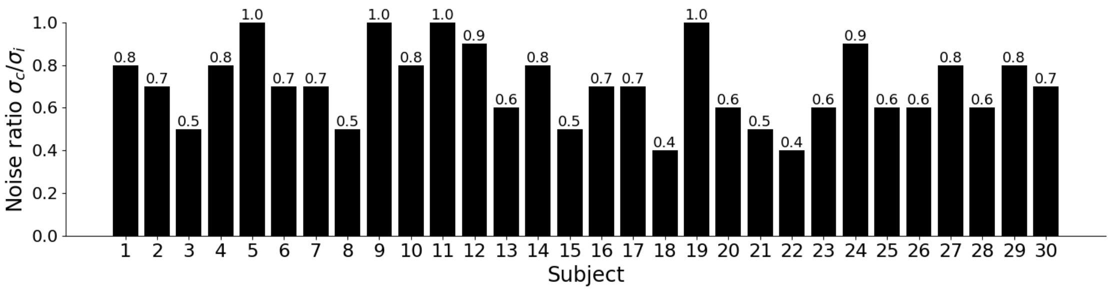

Figure S7: Personalized values of σ_c/σ_i after model personalization

Figure S8 shows the FC matrices obtained from the synthetic BOLD signal for the optimal parameter selection for each subject, compared with the subject's empirical BOLD FC. The optimal value of G and the resulting value of PCC and RMSE between empirical and synthetic matrices is also displayed.

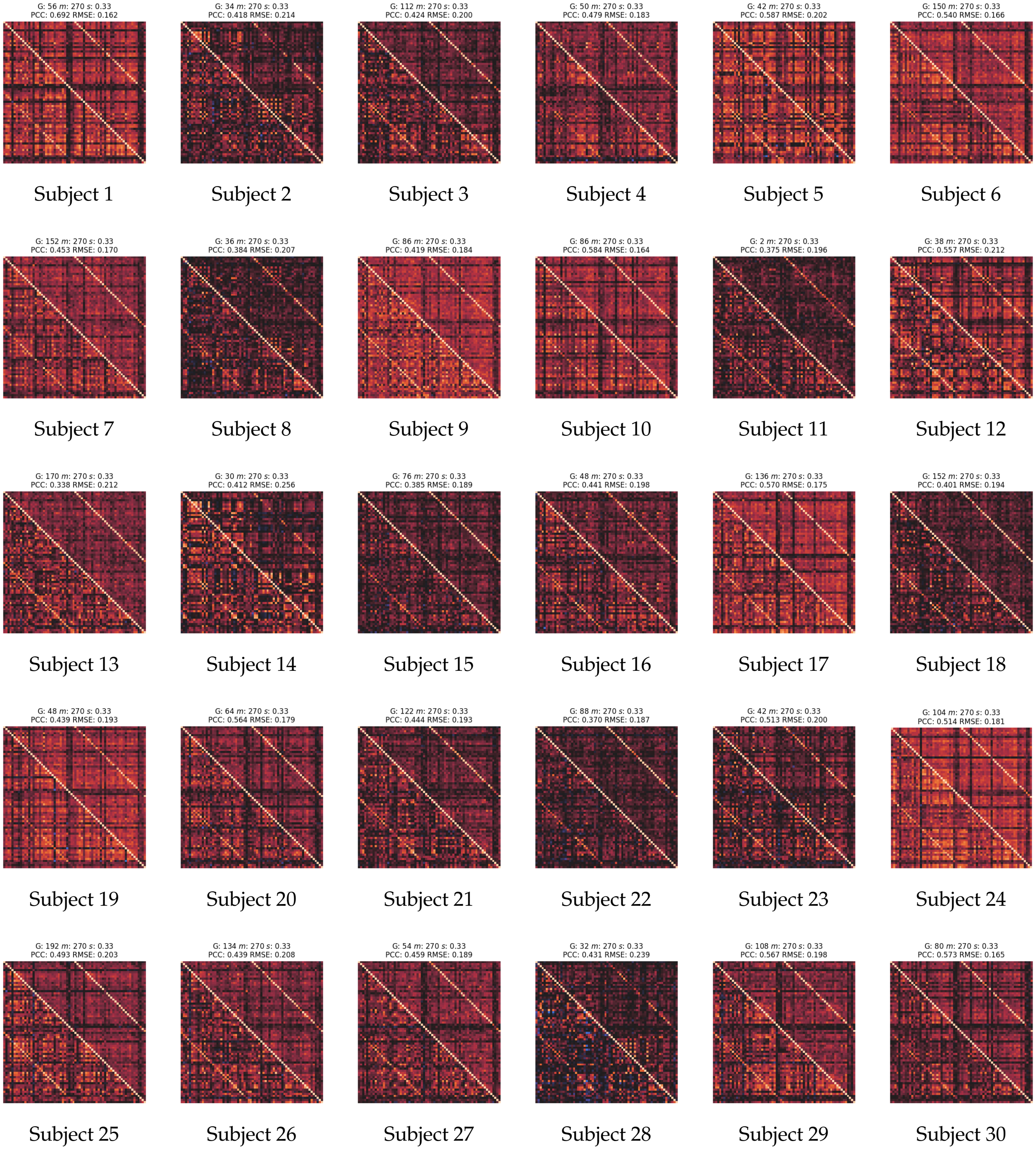

Figure S8: Comparison between the empirical rs-fMRI BOLD FC (lower triangular matrix) and the synthetic FC obtained with the fitted model (upper triangular matrix) for the best set of parameters selected for each subject.

S8 DYNAMIC FC ANALYIS

In order to assess whether the simulated whole-brain models reproduced realistic temporal fluctuations in FC, we computed the dynamic FC (dFC) of the model-generated BOLD signals for each subject. For each simulation (5 minutes in duration), the BOLD time series was divided into 60 non-overlapping sliding windows of 5 seconds. For each window, centered at time t, we computed a separate FC matrix, FC(t). The dFC was then represented as a 60-by-60 symmetric matrix, where each element dFC(t1, t2) corresponds to the Pearson correlation between the upper-triangular elements of FC(t1) and FC(t2). To ensure that the observed temporal variability reflected genuine network dynamics rather than sampling noise, we computed a null distribution by generating surrogate randomized BOLD signals for each patient, which were generated by shuffling the time points of each patient's simulated BOLD data.

For each subject, the variance of the dFC upper triangular matrix entries was higher in the simulated data than in the surrogate control. Figure S9A shows the variance of dynamic functional connectivity (dFC) across subjects for simulated versus random BOLD data, which is significantly higher in the simulated models ($p < 0.001$, paired test), confirming that the model-generated signals exhibit non-trivial temporal fluctuations in functional connectivity beyond those expected from random noise. The mean dFC values further support this result (Figure S9B): while the surrogate data show a mean close to zero, reflecting the absence of cross-temporal correlations, the simulated data exhibit a positive mean, indicating consistent temporal dependencies between FC patterns. As an example, Figure S9C shows the histogram of the upper-triangular dFC values for subject 1, where the simulated dFC exhibits higher variance and mean than for randomized BOLD data.

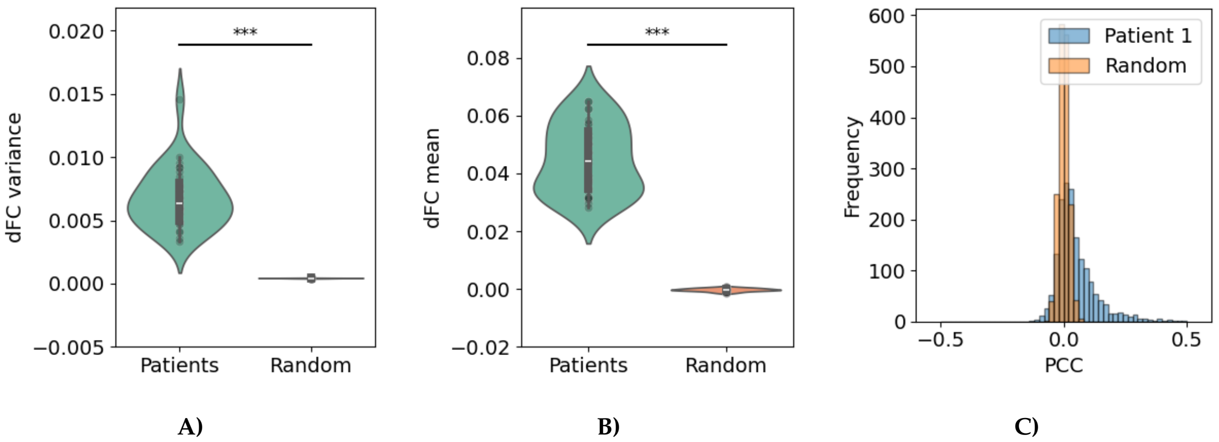

Figure S9: (A) Variance and **(B)** mean of dynamic functional connectivity in simulated versus random BOLD data. Each point represents one subject. The variance and mean of the upper-triangular dFC values was computed for both the simulated BOLD signals and for surrogate random BOLD time series. The violin plots show that the simulated data exhibit substantially higher dFC variance and mean than the random control (***$p < 0.001$, paired test), confirming the presence of non-trivial temporal fluctuations in functional connectivity across time. **(C)** As an example, we show the histogram of the upper-triangular dFC values for subject 1 compared to the random BOLD time series.

S9 PV ACTIVITY UNDER PSYCHEDELICS

Because of the potential involvement of PV interneuron dysfunction in Alzheimer's disease (AD) (Palop & Mucke, 2016), we examined whether simulated PV-like inhibitory activity was enhanced under psychedelics. Although the baseline models used here did not explicitly include PV impairment, as in Sanchez-Todo et al. (2024), this analysis allows us to assess whether 5-HT2A receptor activation indirectly promotes PV-related inhibitory function.

For each subject-specific model, we extracted the simulated PV population time series from both baseline and psychedelic conditions. We then computed (i) the mean PV firing rate, excluding the initial 5 seconds of transient activity, and (ii) the average gamma-band power (30–80 Hz) from the same time series. Both metrics were averaged across cortical parcels for each subject.

As shown in Figure S10, models under psychedelic modulation exhibited a significantly higher mean PV firing rate compared to baseline ($p < 0.001$), although the absolute difference was modest. Similarly, we observed a small but significant increase in gamma-band power ($p < 0.001$), consistent with enhanced fast inhibitory–excitatory interactions.

These results suggest that psychedelic modulation may partly restore or strengthen PV-like inhibitory activity, reflected in both increased firing and enhanced gamma synchronization. Although no explicit PV deficit was introduced here, the observed pattern supports the hypothesis that 5-HT2A receptor activation can rebalance cortical excitatory–inhibitory dynamics via indirect facilitation of PV interneuron function. A more direct test of this compensatory mechanism, incorporating explicit PV impairment (Sanchez-Todo et al., 2024), could be pursued in future work.

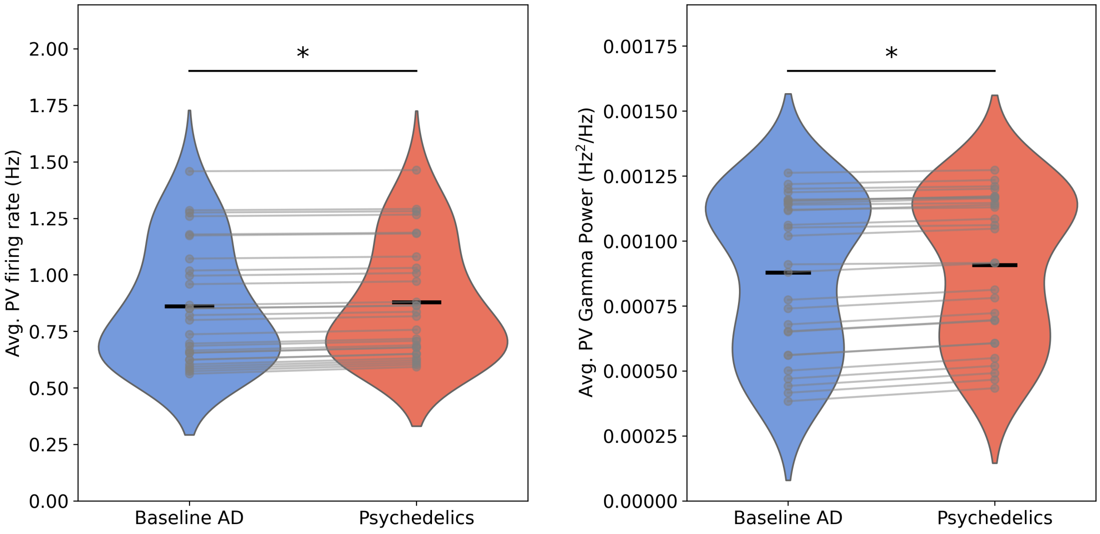

A) **B)**

Figure S10: PV activity under psychedelics (A) Average PV population firing rate across patients in the baseline AD and psychedelic conditions. **(B)** Average PV gamma-band power (30–80 Hz) computed from the same simulated PV time series. Grey dots indicate individual patient averages, horizontal black bars denote medians. The asterisk denotes a significant difference between conditions ($p < 0.001$ in a paired t-test).

REFERENCES

Alzheimer's, A. (2020). 2020 Alzheimer's disease facts and figures. *Alzheimer's & Dementia: The Journal of the Alzheimer's Association*. doi: https://doi.org/10.1002/alz.12068

Babiloni, C., Blinowska, K., Bonanni, L., Cichocki, A., De Haan, W., Del Percio, C., ... Randall, F. (2020). What electrophysiology tells us about alzheimer's disease: a window into the synchronization and connectivity of brain neurons. *Neurobiology of Aging*, *85*, 58–73. doi: https://doi.org/10.1016/j.neurobiolaging.2019.09.008

Bastos, A. M., Usrey, W. M., Adams, R. A., Mangun, G. R., Fries, P., & Friston, K. J. (2012). Canonical microcircuits for predictive coding. *Neuron*, *76*(4), 695–711. doi: https://doi.org/10.1016/j.neuron.2012.10.038

Casula, E. P., Pellicciari, M. C., Bonnì, S., Borghi, I., Maiella, M., Assogna, M., ... Koch, G. (2022). Decreased Frontal Gamma Activity in Alzheimer Disease Patients. *Annals of Neurology*, *92*(3), 464. doi: https://doi.org/10.1002/ana.26444

Fischl, B. (2012). Freesurfer. *Neuroimage*, *62*(2), 774–781. doi: https://doi.org/10.1016/j.neuroimage.2012.01.021

Fischl, B., Van Der Kouwe, A., Destrieux, C., Halgren, E., Ségonne, F., Salat, D. H., ... others (2004). Automatically parcellating the human cerebral cortex. *Cerebral cortex*, *14*(1), 11–22. doi: https://doi.org/10.1093/cercor/bhg087

Garcés, P., Vicente, R., Wibral, M., Pineda Pardo, J. A., Lopez, M. E., Aurtenetxe, S., ... Fernández, A. (2013). Brain-wide slowing of spontaneous alpha rhythms in mild cognitive impairment. *Frontiers in Aging Neuroscience*, *5*. doi: https://doi.org/10.3389/fnagi.2013.00100

Gaubert, S., Raimondo, F., Houot, M., Corsi, M.-C., Naccache, L., Diego Sitt, J., ... Alzheimer's Disease Neuroimaging Initiative (2019). EEG evidence of compensatory mechanisms in preclinical Alzheimer's disease. *Brain*, *142*(7), 2096–2112. doi: https://doi.org/10.1093/brain/awz150

Hansen, J. Y., Shafiei, G., Markello, R. D., Smart, K., Cox, S. M. L., M., N., ... others (2022). Mapping neurotransmitter systems to the structural and functional organization of the human neocortex. *Nature neuroscience*, *25*(11), 1569–1581. doi: https://doi.org/10.1038/s41593-022-01186-3

Jaramillo, J., Mejias, J. F., & Wang, X. (2019). Engagement of pulvino-cortical feedforward and feedback pathways in cognitive computations. *Neuron*, *101*(2), 321–336. doi: https://doi.org/10.1016/j.neuron.2018.11.023

Jurcak, V., Tsuzuki, D., & Dan, I. (2007). 10/20, 10/10, and 10/5 systems revisited: their validity as relative head-surface-based positioning systems. *Neuroimage*, *34*(4), 1600–1611. doi: https://doi.org/10.1016/j.neuroimage.2006.09.024

López, M. E., Bruna, R., Aurtenetxe, S., Pineda-Pardo, J. Á., Marcos, A., Arrazola, J., ... Maestú, F. (2014). Alpha-band hypersynchronization in progressive mild cognitive impairment: a magnetoencephalography study. *Journal of Neuroscience*, *34*(44), 14551–14559.

López-Sanz, D., Bruña, R., Garcés, P., Martín-Buro, M. C., Walter, S., Delgado, M. L., ... Maestú, F. (2017). Functional Connectivity Disruption in Subjective Cognitive Decline and Mild Cognitive Impairment: A Common Pattern of Alterations. *Frontiers in Aging Neuroscience*, *9*, 109. doi: https://doi.org/10.3389/fnagi.2017.00109

López-Sanz, D., Bruña, R., Garcés, P., Camara, C., Serrano, N., Rodríguez-Rojo, I. C., ... others (2016). Alpha band disruption in the AD-continuum starts in the subjective cognitive decline stage: a MEG study. *Scientific reports*, *6*(1), 37685.

Markello, R. D., Hansen, J. Y., Liu, Z., Bazinet, V., Shafiei, G., E., S. L., ... others (2022). Neuromaps: structural and functional interpretation of brain maps. *Nature Methods*, *19*(11), 1472–1479. doi: https://doi.org/10.1038/s41592-022-01625-w

Mejias, J. F., Murray, J. D., Kennedy, H., & Wang, X. (2016). Feedforward and feedback frequency-dependent interactions in a large-scale laminar network of the primate cortex. *Science advances*, *2*(11), e1601335. doi: https://doi.org/10.1126/sciadv.1601335

Müller, F., Lenz, C., Dolder, P., Lang, U., Schmidt, A., Liechti, M., & Borgwardt, S. (2017). Increased thalamic resting-state connectivity as a core driver of LSD-induced hallucinations. *Acta Psychiatrica Scandinavica*, *136*(6), 648–657. doi: https://doi.org/10.1111/acps.12818

Murty, D. V., Manikandan, K., Kumar, W. S., Ramesh, R. G., Purokayastha, S., Nagendra, B., ... Ray, S. (2021). Stimulus-induced gamma rhythms are weaker in human elderly with mild cognitive impairment and Alzheimer's disease. *eLife*, *10*, e61666. (Publisher: eLife Sciences Publications, Ltd) doi: https://doi.org/10.7554/eLife.61666

Nakamura, A., Cuesta, P., Fernández, A., Arahata, Y., Iwata, K., Kuratsubo, I., ... Kato, T. (2018). Electromagnetic signatures of the preclinical and prodromal stages of Alzheimer's disease. *Brain: A Journal of Neurology*, *141*(5), 1470–1485. doi: https://doi.org/10.1093/brain/awy044

Palop, J. J., & Mucke, L. (2016). Network abnormalities and interneuron dysfunction in Alzheimer disease. *Nature Reviews. Neuroscience*, *17*(12), 777–792.

doi: https://doi.org/10.1038/nrn.2016.141

Passero, S., Rocchi, R., Vatti, G., Burgalassi, L., & Battistini, N. (1995). Quantitative EEG mapping, regional cerebral blood flow, and neuropsychological function in Alzheimer's disease. *Dementia (Basel, Switzerland)*, *6*(3), 148–156. doi: https://doi.org/10.1159/000106938

Petersen, R. C. (2004). Mild cognitive impairment as a diagnostic entity. *Journal of Internal Medicine*, *256*(3), 183–194. doi: https://doi.org/10.1111/j.1365-2796.2004.01388.x

Puttaert, D., Wens, V., Fery, P., Rovai, A., Trotta, N., Coquelet, N., ... De, X., Tiège (2021). Decreased Alpha Peak Frequency Is Linked to Episodic Memory Impairment in Pathological Aging. *Frontiers in Aging Neuroscience*, *13*. doi: https://doi.org/10.3389/fnagi.2021.711375

Rochart, R., Liu, Q., Fonteh, A. N., Harrington, M. G., & Arakaki, X. (2020). Compromised behavior and gamma power during working memory in cognitively healthy individuals with abnormal CSF amyloid/tau. *Frontiers in Aging Neuroscience*, *12*, 574214.

Ronneberger, O., Fischer, P., & Brox, T. (2015). U-net: Convolutional networks for biomedical image segmentation. In N. Navab, J. Hornegger, W. M. Wells, & A. F. Frangi (Eds.), *Medical image computing and computer-assisted intervention – miccai 2015* (pp. 234–241). Cham: Springer International Publishing.

Ruffini, G. (2015). Application of the reciprocity theorem to EEG inversion and optimization of EEG-driven transcranial current stimulation (tcs, including tdcs, tacs, trns). *arXiv*. doi: https://doi.org/10.48550/arXiv.1506.04835

Salvador, R., Biagi, M., Pelegrí, M. P., Zhou, J., Travison, T., Pascual-Leone, A., ... Ruffini, G. (2022). Towards the identification and optimization of the "dose-response" relationship of transcranial direct current stimulation. *bioRxiv*, 2022–01.

Sanchez-Todo, R., Lopez-Sola, E., Mercadal, B., & Ruffini, G. (2024). Laminar Neural Mass Model for Representing Alzheimer's Disease Electrophysiopathology. *in prep*.

Sanchez-Todo, R., & Bastos, A. M. e. a. (2023). A physical neural mass model framework for the analysis of oscillatory generators from laminar electrophysiological recordings. *Neuroimage*, *270*, 119938. doi: https://doi.org/10.1016/j.neuroimage.2023.119938

Sperling, R. A., Aisen, P. S., Beckett, L. A., Bennett, D. A., Craft, S., Fagan, A. M., ... others (2011). Toward defining the preclinical stages of alzheimer's disease: Recommendations from the national institute on aging-alzheimer's association workgroups on diagnostic guidelines for alzheimer's disease. *Alzheimer's & dementia*, *7*(3), 280–292. doi: https://doi.org/10.1016/j.jalz.2011.03.003

Uddin, L. Q., Mooshagian, E., Zaidel, E., Scheres, A., Margulies, D. S., Kelly, A. M. C., ... others (2008). Residual functional connectivity in the split-brain revealed with resting-state functional MRI. *Neuroreport*, *19*(7), 703–709. doi: https://doi.org/10.1097/WNR.0b013e3282fb8203

Wang, X., Leong, A. T. L., Chan, R. W., Liu, Y., & Wu, E. X. (2019). Thalamic low frequency activity facilitates resting-state cortical interhemispheric MRI functional connectivity. *Neuroimage*, *201*, 115985. doi: https://doi.org/10.1016/j.neuroimage.2019.06.063
